# Interventions to prevent suicide and suicidal thoughts or behaviours in the perinatal period: a global mixed-methods systematic review

**DOI:** 10.1016/j.eclinm.2026.104318

**Published:** 2026-11-09

**Authors:** Cristina Fernandez Turienzo, Yagmur Diana Erpolat, Kaat De Backer, Sam Burton, Hannah Rayment-Jones, Claire A. Wilson, Jane Sandall, Abigail Easter

**Affiliations:** a Faculty of Life Sciences & Medicine, Department of Women and Children's Health, King's College London, London, United Kingdom; b School of Psychology, Liverpool John Moores University, Liverpool, United Kingdom; c Institute of Psychiatry, Psychology & Neuroscience, King's College London, London, United Kingdom; d South London and Maudsley NHS Foundation Trust, London, United Kingdom

**Keywords:** Pregnancy, Postpartum, Maternal mortality, Perinatal suicide, Suicidality, Prevention

## Abstract

**Background:**

Suicide is a leading cause of maternal death during the perinatal period. While routine enquiry and identification is widely recommended, evidence on comprehensive interventions remains limited. This review evaluated global evidence on the effectiveness of interventions for perinatal suicide, associated thoughts and behaviours, and key implementation features.

**Methods:**

We conducted a mixed-methods systematic review using the Joanna Briggs Institute methodology. We searched eight databases and other sources from inception to December 3, 2025, for global evidence across all income settings without language restrictions. We included studies evaluating interventions, programmes or strategies reporting on our primary outcome: perinatal suicide or suicidal thoughts or behaviours (including ideation, self-harm, attempts) during pregnancy or one year after birth. Data were extracted and appraised for methodological quality. Meta-analysis was not possible due to clinical and methodological heterogeneity; data were synthesised using a convergent integrated approach. PROSPERO: CRD42025625903.

**Findings:**

We screened 27,045 records; 19 records representing nine studies were included: three randomised controlled trials, one secondary analysis of a randomised controlled trial, one non-randomised controlled trial, one quasi-experimental study, one cross-sectional descriptive study, one economic modelling, and one qualitative study. Studies were conducted between 2011 and 2025 in North America, Europe and Asia and included 3613 participants from at risk or disadvantaged populations across eight studies, plus a simulated cohort of 1000. We found trials of indirect clinical interventions targeting parenting or attachment did not reduce suicidality, whereas task-shared behavioural activation psychotherapy targeting depressive drivers, was associated with an 80% reduction in odds of suicidal ideation at three months in a secondary trial analysis. System or community-level strategies in three trials and the modelling were associated with reduced suicide or suicidal ideation by integrating routine screening within multidisciplinary pathways that ensured immediate linkage to care. Informal or crisis support mechanisms facilitated immediate access and worked as protective buffers against self-harm in two feasibility and qualitative studies; faith practices helped counter barriers to formal care while helplines remained limited by them.

**Interpretation:**

Our results suggest that suicide prevention could be improved by embedding routine identification within coordinated health and community safety nets ensuring immediate linkage to care. Expanding evidence-based psychological care and task-sharing targeting depressive drivers of suicidality may help at-risk women receive timely support. Future research explicitly designed and powered for perinatal suicide prevention is needed.

**Funding:**

The 10.13039/501100000272National Institute for Health and Care Research.

**Research in context**

**Evidence before this study**

Suicide is a leading cause of maternal mortality globally, with recent data showing rising rates of suicidal ideation during the perinatal period. We searched two databases (EMBASE, PubMed) from inception to September 7, 2025, using terms related to pregnancy, the postpartum period, suicide, self-harm, and prevention, for global evidence on interventions that reported outcomes and implementation factors related to perinatal suicide or suicidal thoughts and behaviours. Previous research has historically focused on estimating prevalence and identifying risk factors rather than evaluating the effectiveness of prevention strategies. While routine enquiry is widely recommended, there remains a lack of comprehensive evidence regarding which interventions reduce suicidal ideation and behaviours in this population.

**Added value of this study**

To our knowledge, this is the first global mixed-methods systematic review to synthesise interventions reporting on suicide and suicidal thoughts or behaviours in the perinatal period. We found standard programmes focused on indirect factors like parenting or attachment did not reduce suicidality, whereas task-shared behavioural activation psychotherapy was associated with an important reduction in suicidal ideation. Our findings also suggest that community and wide system strategies that integrate routine identification with safety nets that ensure immediate access to care, could reduce suicide. We identified the potential role of informal crisis support systems, such as faith-based practices and helplines, in bridging gaps for populations facing structural barriers.

**Implications of all the available evidence**

Preventing perinatal suicide requires coordinated, stratified health and community systems that can respond to structural barriers. Clinical efforts should consider integrating targeted, evidence-based psychological therapies that address the mechanistic drivers of suicidality, alongside informal, culturally relevant community support. Task-sharing these therapies with non-specialists shows promise as a scalable solution. Future research should evaluate these interventions in large-scale population studies or trials using validated, suicide-specific outcomes. Adopting a life-course approach to maternal suicide prevention through integrated mental healthcare and extended postpartum support is also needed.

## Introduction

Suicide is a leading cause of mortality during pregnancy and in the first year after childbirth (the perinatal period), accounting for 20% of global postpartum deaths.^1^, ^2^ It is also a leading cause of death among women of reproductive age, with 132,000 deaths of women aged 15–49 years occurring globally in 2023.^3^ Suicidal ideation, defined as thoughts of ending one's own life, affects an estimated 8% of individuals globally during the perinatal period^4^, ^5^ and predicts later suicidal behaviour including attempted suicide and suicide.^5^ In the United States, suicidal ideation during the perinatal period increased by 100% between 2008 and 2018, with wide ethnic disparities including a disproportionate 700% increase among Black individuals.^6^ Sub-Saharan African countries face suicidal ideation rates of 15–33% among perinatal individuals, nearly triple those in Western Europe or Australia.^3^ Recent UK data further underscore this crisis, with a 55% increase in postpartum ideation reported since the COVID-19 pandemic.^7^, ^8^, ^9^ Beyond the immediate loss of life, perinatal suicide carries a significant burden to families and societies, impacting child development and generating an intergenerational cycle of psychiatric morbidity.^10^ Addressing this public health crisis is central to Sustainable Development Goal 3.4, which aims to reduce premature mortality and promote mental health and wellbeing globally.

To date, research on perinatal suicide has historically focused on estimating prevalence and identifying risk factors.^11^ Mental disorders, including severe psychotic disorders and common mental disorders (e.g., depression and anxiety) are among the most common complications in childbirth, affecting nearly one in five perinatal individuals, and when poorly managed, are associated with worse outcomes.^12^ Although the presence of mental disorders is among the strongest predictors of suicidal thoughts and behaviours, risk is multifaceted and influenced by a range of interacting factors. These include trauma (e.g., childhood abuse, intimate partner violence, conflict), socioeconomic disadvantage (e.g., young age, poverty, low education, Black ethnicity), and psychosocial stressors (e.g., stress, social isolation, drug misuse, sleep deprivation, death of close person).^12^, ^13^, ^14^, ^15^, ^16^ While understanding the epidemiology and risk factors for perinatal suicide is essential, urgent public health action is needed to address what remains a leading contributor to maternal mortality.^11^ Advances in both pharmacological and non-pharmacological treatments for perinatal mental disorders have improved identification and management of mental health disorders in the perinatal period. However, evidence on effective strategies to prevent suicide among perinatal populations remains limited and this has also been highlighted in England's latest suicide prevention strategy which includes pregnant women and new mothers as a priority group.^17^ Maternal health systems have a unique, often lost opportunity to intervene during pregnancy and the first year after childbirth, as women are in frequent contact with health services for both themselves and their children. And given the drivers for perinatal suicide extend beyond the presence of mental disorder alone, there is a need for targeted evidence-based, holistic approaches that also align with the World Health Organisation's guideline for integration of perinatal mental health into maternal and child health services.^18^, ^19^

Suicide is frequently considered within a broader continuum of suicidal thoughts and behaviour, ranging from suicidal ideation and planning to suicide attempts and death. This continuum reflects the complex and interacting social, structural, and individual determinants that shape suicide risk over time.^20^ Suicide prevention frameworks often therefore adopt a public health approach, recognising the need for coordinated action across multiple levels, including universal population strategies, selective interventions for groups at increased risk, and indicated interventions for individuals experiencing suicidal thoughts or behaviours.^21^ Within these frameworks, outcomes such as suicidal ideation, self-harm, and suicide attempts represent earlier stages along the pathway to suicide and are therefore important targets for prevention research. Since suicide is a relatively rare outcome, research evaluating prevention strategies often examined this broader spectrum of suicidal thoughts and behaviours.^22^ However, it remains unclear how such prevention approaches have been applied within the perinatal period or how effective they are for this population. We therefore aimed to evaluate interventions, programmes, and strategies reporting prevention or reduction of suicide and suicidal thoughts or behaviours during the perinatal period, and identify key features associated with effective prevention and the reduction of inequalities.

## Methods

A global mixed-methods systematic review was undertaken to assess the effect of existing interventions for prevention or reduction of suicide, and suicidal thoughts and behaviours (including suicidal ideation, self-harm, and suicide attempts) during the perinatal period (e.g., pregnancy to one year postpartum), and identify key features and components that might contribute to effective implementation. Anticipating a scarcity of trials directly addressing perinatal suicide and need to explore implementation factors, we followed the Joanna Briggs Institute (JBI) methodological guidance for the conduct of mixed-methods systematic reviews, which allows for the combination of evidence from quantitative, qualitative, and mixed-methods studies into a single cohesive narrative considering limited evidence in this field.^23^ This review was registered with PROSPERO (CRD42025625903) and followed PRISMA reporting guidelines.^24^

### Search strategy and selection criteria

In this global review, we assessed studies that reported any intervention on perinatal suicide or suicidal thoughts and behaviours. A preliminary search of PROSPERO, PubMed and the Cochrane Database of Systematic Reviews found no existing or in progress such a review. We searched MEDLINE, Embase, Global Health, PsycINFO, Web of Science, ASSIA, CINAHL and CENTRAL from inception to December 3, 2025. We did not include any country or language restrictions. Our search strategy combined a comprehensive list of maternal suicide and suicidality related terms (see Appendix 1, pp 1–3). We also hand-searched bibliography and reference lists of the studies identified and other reviews, systematic reviews and meta-analyses, relevant websites, national and international reports and guidelines and dissertations and thesis; Google Scholar, Open Grey, the ISRCTN registry, and the Australian and New Zealand Clinical Trials Registry.

Studies were eligible if they evaluated an intervention, programme or strategy delivered during pregnancy or up to one year postpartum, and reported outcomes (whether primary or secondary) related to suicide or suicidal thoughts and behaviours. Given that suicide is a rare outcome in intervention studies, and is often conceptualised as a spectrum of thoughts and behaviours, we adopted a broad outcomes framework. Therefore, studies reporting on suicide, suicide attempts, self-harm or suicidal ideation were eligible for inclusion. Quantitative measures of these outcomes included event rates (e.g., incidence of self-harm, suicide), effect estimates (e.g., odds ratios, 95% CIs), and validated measures of suicidal ideation (e.g., scores >0 on the Edinburgh Postnatal Depression Scale [EPDS] Item 10 ‘The thought of harming myself has occurred to me’). For qualitative and mixed-methods evidence, outcome data comprised verbatim participant reports, implementation features, and perceived protective mechanisms against self-harm. Studies were included regardless of their study design; restricting the review to trials alone might have excluded potentially relevant studies (e.g., system/population-level, target trial emulation and economic modelling, which are valuable for projecting population-level impacts given the rarity of perinatal suicide as a primary outcome in traditional trials). We excluded studies if they only reported prevalence rates, studied risk factors without an intervention, presented no intervention or did not report on suicide or suicide thoughts or behaviours (see Appendix 1, p 1 for detailed inclusion and exclusion criteria). We did not include interventions targeting women before pregnancy or beyond one year postpartum, All records were uploaded to Covidence software and duplicates removed. Titles, abstracts, and full texts (including associated secondary and exploratory papers) were screened by two independent reviewers (CFT, YDE, KDB, AE) and discrepancies were resolved by consensus or referred to a third reviewer.

### Data extraction and appraisal

We developed and piloted a data extraction form covering six broad areas: 1) characteristics of study type and settings country, year, design including randomised trial, quasi or non-randomised trial, quantitative, qualitative or mixed methods, primary aim, setting (community, healthcare institutions, industry) and ethics; 2) the description of study participants, including inclusion and exclusion criteria, total numbers, mean age, ethnicity, timing, marital status, education and socio-economic status, pre-existing medical or mental health conditions and other lifestyle/health behaviours such us substance misuse, physical/sexual abuse or previous suicide attempts; 3) descriptions of interventions (and comparison if any) and its components such as what was provided, who provided it, how and where it was delivered, and the dosage or frequency; 4) description of outcome measures (effect size, numbers, percentages, quotes); 5) description of how the intervention may work (theory of change); 6) and descriptions of intervention challenges and strategies, with a blank field for other details that were deemed relevant to perinatal suicide. The data extraction form also integrated the Mixed Methods Appraisal Tool (MMAT) to assess methodological quality of any study type concurrently with data collection.^21^ JBI and MMAT guidelines discourage the provision of numerical quality scores and instead recommend a narrative summary. The extraction template and raw extraction data are included in Appendix 1 (pp 4–30). Each extraction was carried out independently by two reviewers (CFT, YDE). A third reviewer (KDB) selected a random sample of extracted data to ensure accuracy and harmonisation.

### Statistical analysis

After data extraction and appraisal, studies underwent further review to discuss the possibility of a meta-analysis to assess the effect of the interventions on maternal suicide and suicidality, but this was not possible given the significant heterogeneity in study designs, interventions and outcomes. We specifically evaluated the feasibility of performing a meta-analysis across four studies reporting suicidal ideation via EPDS Item 10.^25^, ^26^, ^27^, ^28^ However, quantitative pooling was not possible due to substantial clinical, methodological, and design heterogeneity (see Appendix 1, p 31). Therefore, we used the JBI's convergent integrated approach to synthetise findings across all study designs. This approach was chosen because both the quantitative data (effectiveness) and qualitative data (implementation processes) addressed our review objective: understanding what works and why. Consequently, data were not segregated into separate summaries, but integrated thematically based on similarity of meaning. Quantitative data regarding suicide and suicidal ideation and behaviour were extracted independently by two reviewers. To ensure comparability with qualitative evidence, we performed ‘qualitisation’, wherein numerical results were systematically converted into narrative, textual statements describing the intervention's effectiveness, while retaining all underlying numerical and statistical metrics (e.g., percentages, odds ratios, 95% CIs, p-values) to preserve complete data granularity. This process was supported by a data conversion table (Appendix pp 31 and 32) which provides an audit trail mapping statistical results to these descriptive statements. Qualitative data, including implementation features, experiences and theories of change, were synthesised using thematic synthesis. Following the initial independent synthesis of the qualitative and qualitised quantitative streams, we integrated these data through a repeated, detailed examination of the pooled findings. We identified key categories based on similarity of meaning, allowing us to combine quantitative evidence of effectiveness with qualitative evidence regarding what works and why (implementation features and theory of change). These categories were finally aggregated into overall integrated findings to address the aim of the review (see synthesis matrix of this integration in Appendix pp 33 and 34).

### Ethics

As this study is a systematic review of publicly available published literature, ethics approval and participant informed consent were not required.

### Role of the funding source

The funder had no role in study design, data collection, data analysis, data interpretation, writing of the report, or the decision to submit the manuscript for publication.

## Results

The searches produced 27,045 records, of which 4594 were removed as duplicates. A total of 22,451 records were screened by title and abstract, and 97 full-text articles. Nineteen records representing nine studies were included.^25^, ^26^, ^27^, ^28^, ^29^, ^30^, ^31^, ^32^, ^33^ All studies were published between 2011 and 2025 and represented a mix of high- and low-income settings and included a wide variety of methods. Three randomised controlled trials were conducted in Europe (Germany, the UK, France),^25^, ^31^, ^32^ one secondary analysis of a randomised controlled trial in the US and Canada,^26^ one non-randomised controlled trial in Japan,^28^ one cross-sectional descriptive study in India^30^; and three additional studies in the USA: one quasi-experimental cross-sectional study,^27^ a hypothetical economic modelling study^33^ and a qualitative study^.^^29^ Fig. 1 summarises the search results and screening process, Table 1 reports the characteristics of included studies (detailed further in Appendix 1, pp 4–30), and a dedicated summary of studies reporting suicidal ideation via EPDS Item 10 is provided in Appendix 1 (p 31). Eight studies included a total of 3613 participants, with an additional simulated cohort of 1000 individuals from the hypothetical economic model^33^ and often targeted at risk and disadvantaged populations. Three studies specifically included women based on clinical risks of severity: women discharged from a psychiatric mother and baby unit with severe illness (e.g., psychosis or bipolar disorder),^30^ women with scores greater than nine or ten on the EPDS indicating probable depression or high emotional distress,^25^, ^26^ or women identified with clinically significant level psychopathology and bonding difficulties.^31^ Other studies focused on socioeconomic disadvantage, including Black and Latina women,^29^ low-income women eligible for Medicaid^29^, ^33^ or women with multiple social risks such as low education, single parenthood, and immigrant status.^32^ Three studies identified women from hospital birth records (Medicaid beneficiaries), birth notifications,^28^ and modelling cohorts^33^ to capture population-level data.

**Fig. 1:**
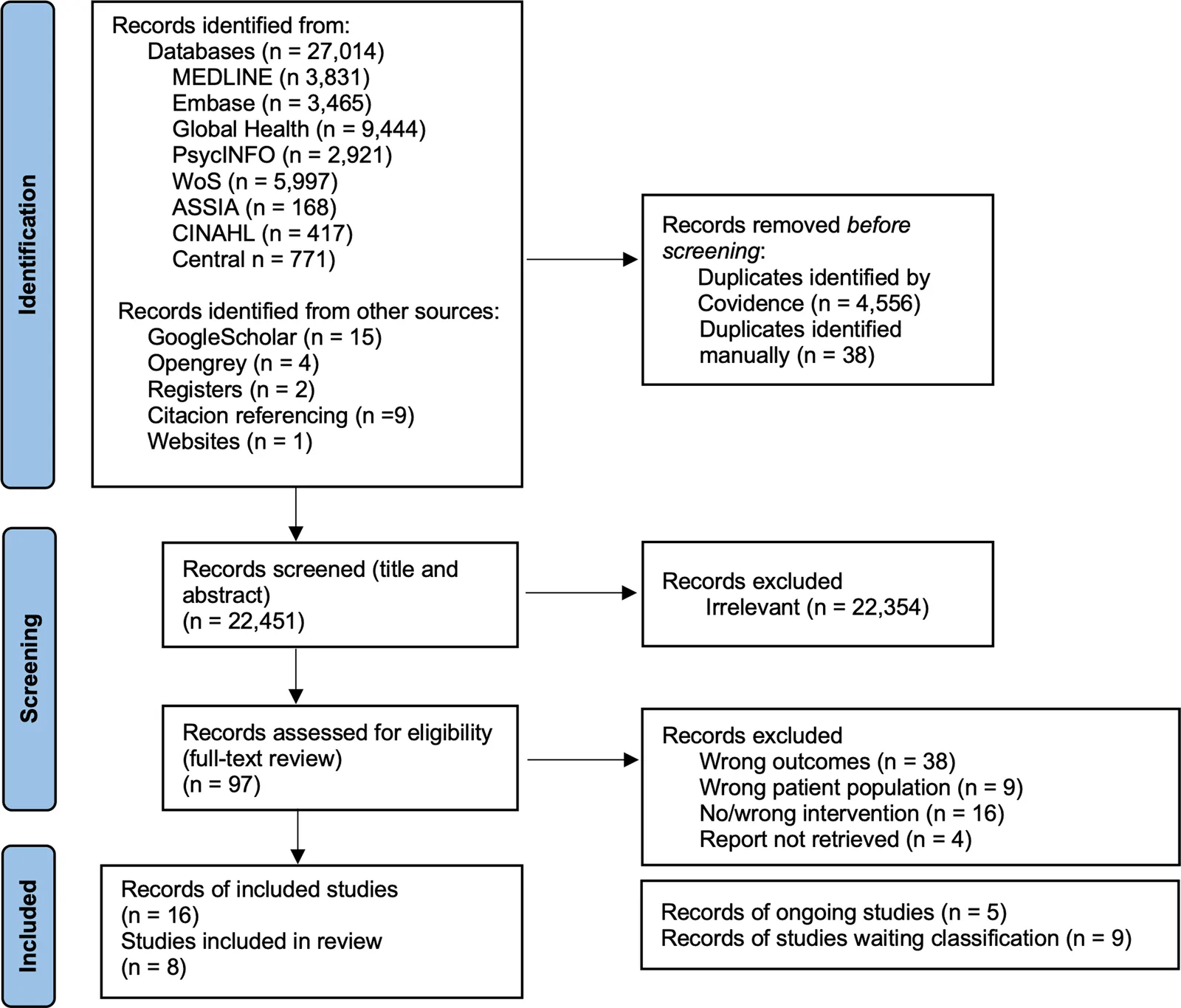
Study profile.

**Table 1:** Characteristics of included studies.

Study ID	Country	Design	Aim	Population	Intervention	Comparator	Theory of change	Suicide related outcomes
Hassdent-eufel 2023^25^	Germany	RCT	To investigate effectiveness of an electronic mindfulness-based intervention (eMBI) on mental health outcomes in pregnant women screened for emotional distress	460 pregnant women (230 intervention; 230 control) who screened positive for mental distress (EPDS >9); mean age ∼33; commonly married (66%), of German origin (85%), and well-educated	‘Mindmom’ an 8-week supervised smartphone-based program combining psychoeducation and cognitive behavioural therapy approaches with mindfulness exercises to target birth anxieties. Delivered via weekly 45-min sessions using audio files, videos, and interactive worksheets (including a personal skills box)	Treatment as usual combined with access to the study app (restricted to completing questionnaires and reading a generic pregnancy guide)	A digital intervention incorporating mindfulness and CBT can help women identify “vicious circles” of fear regarding birth and parenting. By acquiring skills like mindful breathing, women can reduce pregnancy-specific anxiety and prevent the onset of postpartum depression.	Suicidal ideation: score >0 on EPDS Item 10 *(adverse event/safety monitoring)* 9/142 (6.3%) in the intervention group vs 20/174 (11.5%) in control group. No statistically significant differences (χ2 = 2.49, p = 0.11).
Rosan 2025^31^	UK	RCT	To evaluate the clinical effectiveness of the Circle of Security-Parenting (COS–P) intervention combined with treatment-as-usual in reducing psychopathology	371 birthing parents (248 intervention; 123 control) with infants <1-year, mean age 30.8; predominantly White (89%), living with a partner (77%), and presenting with depression (81%) or anxiety (81%) alongside bonding difficulties	Circle of Security-Parenting (COS–P): A 10-week, attachment-informed group program delivered via a hybrid model (predominantly online) by trained psychological practitioners, using a specific perinatally adapted manual and video reflections to help parents discuss their own attachment experiences and improve their ability to recognise and respond to their infant's emotional cues	Treatment-as-usual: standard NHS care tailored to clinical needs, which could include psychiatric reviews, pharmacological interventions, psychological therapies (e.g., CBT), and parent–infant interventions.	By targeting emotion regulation and reflective functioning (understanding the infant's needs) in a supportive group, the intervention aims to improve parent-infant bonding, thereby reducing parental psychopathology and optimising child development.	Self-harm or concerns about self-harm *(adverse event/safety monitoring)* 11 (3%) participants in total: 9 in intervention 2 in control)
Saïas 2013^32^	France	RCT	To assess the impact of a home-visiting program on child mental health, postnatal depression, and quality of the home environment.	440 vulnerable women (222 intervention; 218 control) Median age: 22.3; predominantly low-income with 46.8% eligible or government welfare; 52.1% first-generation immigrants 83.9% low educational attainment and less than 12 years of schooling; 44.2% living alone; 44.7% of presenting depressive	A manualized and tailored home-visiting program delivered by trained clinical psychologists, consisting of 44 visits scheduled from the third trimester of pregnancy until the child's second birthday. Strategies included the use of video feedback to enhance maternal sensitivity and specific guidance on infant emotional development to promote secure attachment.	Usual care within French health system, including free access to community mental health centres and mother-child protection services, in addition to seven home-based assessment visits by independent evaluators.	Bronfenbrenner's ecological model, Bowlby's attachment theory, and Bandura's self-efficacy theory to mitigate the impact of accumulated environmental risk factors. Hypothesised that by empowering mothers and addressing past traumas, the program improves maternal sensitivity and secures infant's long-term mental health	Suicide attempts *(adverse event/safety monitoring)* 0 in intervention 0 in control
Kaliush 2025^26^	USA & Canada	Secondary analysis of an RCT	To determine whether the likelihood of endorsing suicide ideation changed during a brief behavioural activation (BA) psychotherapy irrespective of clinician or delivery types.	1117 pregnant (36 weeks) and postpartum (4–30 weeks) adults with depressive symptoms (EPDS 10). Mean age 33.4; 50% White; highly educated (40% graduate degrees). 23.6% endorsed SI during treatment.	Brief BA, a manualized 6–8 session weekly psychotherapy focussing on increasing values-consistent living and awareness of triggers. Delivered in four arms to test task-sharing: by specialists (psychologists, psychiatrists, social workers) vs non-specialists (nurses, midwives), and via telemedicine vs in-person.	Active comparators: comparison between delivery person and methods. No non-intervention control group.	BA reduces suicide risk by increasing values-consistent living and awareness of ineffective behaviours. By helping patients identify triggers for depressive thoughts and reconnect with core values, it alleviates hopelessness and key driver of suicidality.	Suicidal ideation: score >0 on EPDS Item 10 *(secondary outcome)* With each additional session (OR, 0.75; 95% CI, 0.58–0.96), and at 3 months (OR, 0.20; 95% CI, 0.14–0.27) No significant difference between provider or delivery modality.
Liepman 2011^27^	USA	Quasi-experimental	To evaluate a community-wide educational and advocacy intervention to improve care for perinatal mood disorders, promote screening and treatment, and enhancing community awareness.	Two representative, random 10% samples of hospitalised postpartum women (N = 658) recruited at two time points: a baseline cohort in 2002 (n = 332) and a post-intervention cohort in 2009 (n = 326).	A comprehensive community-wide initiative that combined the training of nearly 500 healthcare providers in screening and referral with a mass media public awareness campaign (18 months). Structurally, the program established a specialised Women's Behavioural Health Clinic to provide expert consultation for prenatal providers and evaluation of complex cases.	Usual care: a historical baseline cohort representing standard community care prior to the implementation of the educational and advocacy program	Community education and advocacy alter healthcare provider behaviours to standardise screening and increase referrals; this increased access to professional help serves as the primary mechanism for reducing depression and suicide risk.	Suicidal ideation: score >0 on EPDS Item 10 *(secondary outcome)* The percentage of women reporting suicidal thoughts significantly decreased from 7.1% in the baseline cohort to 3.5% in the post-intervention cohort. (EPDS Q10 score).
Tachibana 2020^28^	Japan	Non-randomised controlled trial	To examine whether an integrated community mental healthcare program could reduce postnatal suicidal ideation and improve maternal mental health	464 postnatal women (mean age ∼32 years) recruited upon birth notification submission. Two cohorts: 234 in historical control group, and 230 in intervention group.	Usual care plus a suicide prevention program comprising 3 components: 1) routine mental health screening (EPDS) by public health nurses during neonatal home visits; 2) case management using “TALK” principles (Tell, Ask, Listen, Keep safe) for those with ideation; and 3) support from a multidisciplinary clinical network (psychiatrists, obstetricians, etc.)	Usual postnatal care alone, consisting of standard home visits by public health nurses without specific mental health screening or the suicide prevention protocol	Routine screening by trusted nurses facilitates opening the conversation and early detection while structured case management ensures accurate assessment, psychoeducation, multidisciplinary support to prevent self-harm recurrence.	Suicidal ideation: score >0 on EPDS item 10 *(primary outcome)* Significantly lower at time 1 (3-4-four months postnatal): Control: 27 (11.5%) Intervention: 10 (4.3%) (p = 0.014) No sig difference at time 2 (p = 0.111).
Ragesh 2020^30^	India	Cross-sectional descriptive	to assess the feasibility, acceptability, usage patterns, & characteristics of a free mobile phone helpline provided to mothers & families discharged from a Mother-Baby Unit	113 mothers from a Mother-Baby Unit; high clinical severity (acute psychosis/bipolar disorder); predominantly young (mean age ∼25), 62% from rural areas, 50% below poverty line and limited literacy	24-h mobile phone helpline managed by a psychiatric social worker with multidisciplinary team support. The service used standard algorithms and operating procedures to provide post-discharge advice, symptom management, and crisis intervention for mothers and their families.	NA	Mobile technology as a potential bridge to mental health care in low-income settings, facilitating collaborative care by connecting discharged patients to a multidisciplinary team; this could empower women to seek help, ensuring continuity of care despite infrastructure barriers	Helpline utilisation for suicidal Ideation *(feasibility and adverse event monitoring)* 51 women (45%) made 248 calls; and 2 of them (0.8%) were made to seek help for suicidal ideation. Among non-users, one maternal death by suicide (woman did not have a phone, and family member held the helpline number but did not call).
Keefe 2016^29^	USA	Qualitative	To explore how faith, church participation and spiritual practices help new African American and Latina mothers to cope with postpartum depression.	30 new, low-income mothers (19 African American, 11 Latina); Ages 18–44; 11 pregnant; all with postpartum depression; 3 reported thoughts of self-harm or suicide. Mean EPDS 18.75 (severe depressive symptoms).	Faith and church participation. Mothers used active engagement with their faith community and personal spiritual practices (such as prayer and surrendering to a higher power) as an informal support system to manage depressive symptoms, gain emotional validation, and access communal resources	N/A	A biopsychosocial-spiritual framework hypothesises that spiritual beliefs and practices function as active coping mechanisms that relieve stress and facilitate cognitive restructuring, enabling mothers to develop new perspectives on their suffering and potentially prevent self-harm.	Qualitative themes, *(secondary)* Three mothers identified faith as protective factor from suicide; immediate symptom relief: “*There was never a day where that praying did not work … all the sudden your spirit just lift up”* or protective mechanisms (“I *know if I was to hurt myself, I could not ask God for forgiveness*")
Wilkinson 2017^33^	USA	Cost-effectiveness modelling study	to model the cost-effectiveness of screening for and treating postpartum depression (PPD) and postpartum psychosis (PP) compared to the current standard of care.	Hypothetical cohort of 1000 women of reproductive age (18–49) experiencing one live birth, and beneficiaries Medicaid (low-income population)	Routine screening using the EPDS during outpatient visits, followed by treatment (fluoxetine and/or interpersonal therapy) provided by primary care physicians under the mentorship of a psychiatrist (collaborative care model).	Usual care, defined as the absence of routine screening, where the majority of cases remain undetected and untreated.	Routine screening significantly increases the detection of PPD and PP; subsequent treatment (medication or therapy) leads to higher remission rates and improved quality of life (QALYs), thereby reducing adverse outcomes such as suicide and justifying increased healthcare costs	Reduction in perinatal suicides and cost-effectiveness (*primary modelled outcome*) Predicted reduction in absolute number of suicides over 2-year period: 0.58 suicides per 1000 women in the usual care group compared to 0.34 per 1000 women in intervention group (difference of −0.24)

BA, Behavioural Activation; CAPEDP, Compétences parentales et Attachement dans la Petite Enfance: Diminution des Pratiques maltraitantes; CBT, Cognitive Behavioural Therapy; CI, Confidence Interval; COS–P, Circle of Security-Parenting; eMBI, electronic mindfulness-based intervention; EPDS, Edinburgh Postnatal Depression Scale; NHS, National Health Service; OR, Odds Ratio; PP, Postpartum Psychosis; PPD, Postpartum Depression; QALYs, Quality-Adjusted Life Years; RCT, Randomised Controlled Trial; TALK, Tell, Ask, Listen, Keep safe.

The studies varied widely in intervention scope, provider, delivery method and its theory of change, as well as measurement and reporting of perinatal suicide and suicidality, and implementation features, but synthesis integration identified three broad categories based on how interventions were intended to work: 1) system or community level strategies, 2) clinical and digital interventions, and 3) informal or accessible crisis support systems. These categories served a pragmatic organisational purpose for this review, and rather than indicating mutually exclusive categories, they operated as complementary, integrated parts of the care pathway. While system or community strategies largely provided the essential architecture to reach and care for women, the clinical and digital interventions represented the therapeutic content itself.

Three studies (two trials and one quasi-experimental) evaluated system-level or community-wide strategies designed to identify women who scored highly on the EPDS with clear referral and management pathways.^27^, ^28^, ^33^ These interventions aimed to improve identification of mental health risk thorough routine enquiry and defined referral and treatment pathway, and were underpinned by medical and public health models, hypothesising that increasing detection rates via universal enquiry would only reduce symptom severity and mortality risk if aligned with defined pathways to professional care. A comprehensive community-wide initiative in the US trained >500 health and social care providers in detection, enquiry and case ascertainment protocols while establishing a specialist clinic for complex cases which resulted in a statistically significant reduction in the frequency of women reporting suicidal thoughts (defined based on score >0 on EPDS Item 10) from 7.1% in the baseline cohort to 3.5% post-intervention.^27^ The Nagano trial in Japan, included a program integrating routine mental health screening by public health nurses, case management, and multidisciplinary clinical networks and found a significantly lower suicidal ideation (EPDS Item 10 > 0) in the intervention group compared to the control group (usual postnatal care with standard home visits without specific mental health screening protocols) at 3–4 months postpartum (4.3% vs 11.5% respectively; p = 0.014), although differences were not significant at 7–8 months (p = 0.111).^28^ Meanwhile, the economic modelling study of a US Medicaid population projected that routine EPDS use in outpatient settings followed by psychiatrist-mentored pharmacotherapy or interpersonal therapy would be cost-effective, potentially preventing 0.24 suicides per 1000 women compared to usual care.^33^ A key implementation finding across these studies was the importance of effective gatekeepers; screening was only successful when supported by a functional multidisciplinary network able to receive referrals, ensuring women identified as at risk did not fall through the net.

Three trials and a secondary analysis of a trial assessed clinical and digital interventions, including skills-based and psychotherapeutic approaches designed to address individual psychopathology and parenting needs.^25^, ^26^, ^31^, ^32^ Unlike system- or community-level interventions, these were time-limited, curriculum-based programmes grounded in attachment theory, ecological models, or cognitive behavioural principles. Two trials focused on parenting and attachment skills: a 10-week attachment-informed group programme (Circle of Security-Parenting) delivered online and face-to-face by trained psychologists in the UK,^31^ and a high-intensity home-visiting programme (CAPEDP) by psychologists from pregnancy through the child's second birthday in France^32^; and found no reductions in suicidality compared to the control group (standard care tailored to clinical needs, which could include e.g. home-based visits, psychiatric reviews, psychological therapies, parent–infant interventions). The UK trial reported 11 (3%) adverse events involving ‘*self-harm or concerns about self-harm’*, which were higher in the intervention group compared to treatment-as-usual (9 vs 2 respectively)^31^ and the France trial reported ‘*no suicide attempts*’ in either group.^32^ Similarly, a digital mindfulness and CBT application (‘mindmom’) monitored remotely by study staff to target pregnancy-related anxiety in Germany and reported no significant differences in suicidal ideation (EPDS Item 10 > 0) between the intervention and a control group receiving treatment-as-usual alongside a restricted version of the study app (limited to generic pregnancy reading) (6.3% vs 11.5%; χ2 = 2.49, p = 0.11).^25^ In contrast, the secondary analysis of the SUMMIT trial of task sharing and Behavioural Activation (BA), a psychotherapy targeting avoidance and hopelessness, significantly reduced suicidality: the odds of endorsing suicidal ideation decreased by 25% with each additional session (odds ratio [OR], 0.75; 95% CI, 0.58–0.96; p = 0.03) and by 80% at 3 months post randomisation (OR 0.20; 95% CI, 0.14–0.27; p < 0.001).^26^ The effect did not differ between specialist (psychologists, psychiatrists, social workers) and non-specialist providers (nurses, midwives, doulas) or between in-person and telemedicine delivery, though this trial was limited by the use of four active comparators (comparing specialists and delivery methods) without a non-intervention control group.^26^ Both specialist and non-specialist providers received the same competency-based training and were assessed by standardised assessments, with clear clinical safety and escalation protocols. Although attrition remained a challenge across this category, the digital mindfulness app saw a 38% dropout rate and the home-visiting programme reported 50% missing data by 18 months.^25^, ^32^ Retention was high in the BA trial (91% analysed), suggesting that brief, focused psychotherapies delivered by non-specialists via telemedicine are acceptable. However, although task-sharing facilitates access, it should be embedded within escalation processes, as some non-responders with complex trauma required more intensive care.^26^

Finally, two smaller studies (quantitative descriptive and qualitative) examined informal or accessible crisis support systems, designed to overcome barriers to mental health care and grounded in accessibility and biopsychosocial-spiritual frameworks, with one study suggesting that spiritual beliefs could actively support emotion regulation and function as cognitive tools preventing self-harm. In India, a 24-h mobile helpline managed by a psychiatric social worker for women with severe mental illness discharged from a mother-baby unit was found to be feasible and used specifically for “*help to deal with suicidal ideation*” during two of the 248 calls (0.8%).^30^ Implementation barriers to technology and gender inequalities were reported to be fatal a woman at risk died by suicide after being unable to access the helpline because she did not own a mobile phone, despite her family having the number.^30^ Qualitative findings from the USA identified faith as a perceived protective coping mechanism that may buffer suicide risk and support emotional regulation, particularly among women experiencing barriers to formal mental healthcare and African American and Latina mothers. Faith functioned both as an immediate coping strategy through prayer and as a protective mechanism through beliefs that suicide was contrary to God's will preventing self-harm (“*I know if I was to hurt myself, I could not ask God for forgiveness*”), while also providing sometimes rapid symptom relief (“*There was never a day where that praying did not work… all of a sudden your spirit just lift up*”).^29^

Overall methodological quality across the included studies was variable. Among the three primary randomised controlled trials,^25^, ^31^, ^32^ all reported robust randomisation and allocation concealment procedures. Blinding of participants was not feasible due to the nature of the interventions (smartphone app or parenting group), though outcome assessor blinding was achieved in two trials.^31^, ^32^ High attrition was a notable risk of bias, with 36%–50% missing data reported in the digital mindfulness and home-visiting trials.^25^, ^32^ Across these three trials, suicidality was tracked solely as an adverse event or safety monitoring metric rather than a prespecified primary efficacy endpoint. Adherence was also variable; the parenting trial reported a mean attendance of 64% (6.4 out of 10 sessions).^31^ In the secondary analysis of the SUMMIT trial, participant blinding to intervention arm and outcome assessor blinding were both achieved and data completeness was high (91% analysed), but the reported 80% reduction in suicidal ideation (measured as a secondary outcome) was derived using available observed data rather than an intention-to-treat analysis.^26^ While it lacked a non-intervention control arm, a significant dose–response effect was established, with suicidality decreasing by 25% with each additional session attended.^26^

Among the non-randomised study^28^ and the quasi-experimental study^27^ and the descriptive cross-sectional study,^30^ all included representative target samples, with suicidal ideation evaluated as a primary outcome in the non−randomised trial^28^ and a secondary outcome in the quasi-experimental study.^27^ However, methodological limitations included the absence of baseline data in an abstract-only report,^27^ potential temporal confounding due to seasonal variation in birth cohorts (potentially influencing depressive symptoms),^28^ and risk of non-response bias where 33% of the target population was lost to follow-up.^30^ The economic modelling study could not be formally appraised as its projections were based on a simulated hypothetical cohort derived from literature rather than empirical trial data.^33^ In contrast, the qualitative study, despite its small sample, it demonstrated methodological quality with clear coherence between the theoretical framework, data collection, and qualitative interpretation.^29^

## Discussion

This global mixed-methods systematic review evaluated the effects of existing interventions on perinatal suicide and suicidal thoughts and behaviours, and identified key features associated with their implementation. Nine studies, using diverse methodologies and conducted in Europe, North America, and Asia between 2011 and 2025 were included. Target populations largely comprised of women with identifiable risk factors, defined by clinical severity (discharge from psychiatric units, positive scores for distress on psychometric scales) or by socioeconomic disadvantage (poverty, minority ethnic group, immigrant status). Across studies, three broad and complementary categories were identified: system or community-level strategies, clinical or digital interventions, and informal or accessible crisis support. System and community level strategies that combined routine enquiry with structured referral and management pathways showed potential for reducing suicidal thoughts or ideation. Clinical and digital interventions targeting more indirect factors such as attachment, parenting or birth anxiety showed limited effect, whereas targeted psychotherapies addressing depressive symptoms showed greater potential for reducing suicidal thoughts. Informal or accessible crisis support mechanisms, including mobile helplines and faith-based practices, provided immediate, accessible and culturally relevant forms of crisis management and self-harm reduction. The mechanisms by which these interventions were meant to work varied from early detection and referral to care, skills-based and psychotherapeutic approaches grounded in attachment, ecological, and cognitive behavioural theories, and improved emotional regulation and barriers to help-seeking.

The core component in system and community level strategies was not detection alone, but the integration of risk identification with structured referral and management pathways that ensured rapid access to care. These interventions showed the greatest potential for reducing suicidality when detection was embedded within a multidisciplinary safety net. The US community-wide initiative and the Japanese suicide prevention programmes established clear clinical networks linking trained gatekeepers (e.g., public health nurses, social workers) to specialist psychiatric care, reducing provider uncertainty and loss to follow-up among women at risk.^27^, ^28^ The economic modelling similarly indicated that risk identification might only be lifesaving and cost-effective when linked to timely treatment initiation.^33^ These findings align with the concept of safety netting in health systems, which uses overlapping layers of clinical, social, and structural protection to prevent patients at high risk from falling through gaps in care^34^; and reinforces evidence that a strong safety culture and active multidisciplinary feedback loops are vital to reduce maternity mortality.^35^ By shifting from routine enquiry and risk identification alone, to an integrated safety net, such approaches address siloed working, poor communication, and fragmented follow-up, all key contributors to perinatal suicide.^36^ This direction is reflected in NHS England's recent Maternal Care Bundle and the WHO perinatal mental health framework, both of which advocate integrated, community-based models with shared responsibility for risk management.^19^, ^37^

Findings from the clinical and digital trials found a clear distinction in intervention targets. Standardised psychological interventions focussing on broader constructs, such as maternal sensitivity, anxiety, parenting or attachment (e.g., Circle of Security-Parenting, mindfulness, home visiting) did not reduce suicidal ideation. These programmes faced implementation challenges, including high attrition and suicide was indeed monitored only as an adverse event rather than addressed as a clinical outcome.^25^, ^31^, ^32^ This aligns with evidence suggesting that indirect interventions without a specific, targeted suicide-prevention focus do not appear to independently reduce suicidal thoughts.^38^ However, the recent task-sharing and BA trial, which directly addressed avoidance and hopelessness, showed significant reductions in suicidal ideation. In contrast to the parenting and attachment focused interventions identified in this review, BA provides specific tools to disrupt the depressive cycle and may reduce suicide risk by increasing values consistent living and awareness of unhelpful behaviours.^39^ A recent review highlighted indeed the transdiagnostic potential of BA, emotion regulation, and cognitive restructuring as core mechanisms for perinatal mental health interventions.^40^ In the BA trial, non-specialists delivered the intervention as effectively as trained specialists, aligning with global evidence on the scalability of task-shared treatments for perinatal mental health including the Lancet Commission on Global Mental Health and WHO guidance on integrated perinatal mental health services.^19^, ^41^, ^42^ And such competency-based training protocols are not limited to this single trial but are successfully leveraged across broader task-sharing platforms globally to build mental health capacity.^43^ These findings do not suggest that parenting or attachment based interventions are unimportant, but rather that interventions seeking to reduce suicidal thoughts may require an explicit focus on the mechanisms underpinning suicidality, alongside broader support for the mother-infant relationship.

An interesting finding of this review is the potential role of informal or accessible crisis support systems to bridging the gap to care for populations facing structural barriers, although evidence was limited to two smaller studies. Qualitative findings showed how faith and church participation could function as a protective mechanism to suicide among ethnic minority groups who may distrust formal systems. Several studies indicate a protective association between religiosity or spirituality and depression or suicide risk in the general population, partly mediated through social support,^43^ with emerging evidence suggesting lower perinatal anxiety and depressive symptoms among those engaging in faith practices.^44^, ^45^ However, no studies have directly assessed this relationship or the impact of faith or organised religious participation on perinatal suicide. Similarly, the 24-h helpline shows the potential of using remote connections for crisis management, providing women had safe access to a mobile phone. However, if this support system is not readily accessible in a crisis, the protective mechanism fails. These findings should not be interpreted as evidence that faith-based practices are clinically effective interventions for reducing suicidality. Rather, they highlight the potential role of informal, culturally relevant coping and support mechanisms that may complement evidence-based clinical care, particularly for women facing structural barriers to accessing services.

This is to our knowledge, the first mixed-methods systematic review to synthesise global interventions, strategies and programmes to prevent perinatal suicide, and related thoughts or behaviours. The main strengths of the study include the robust JBI methodology, the inclusivity of diverse study designs, interventions and outcomes across diverse settings, despite no study from Africa or Latin America, and the integration of a large trial which provides high-quality evidence on task-sharing and BA. Statistical inter-rater reliability coefficients were not calculated as per JBI, and coding alignment was managed through parallel independent extraction and consensus with a third reviewer. The heterogeneity of study designs, interventions and reported outcomes was a limitation and made a meta-analysis impossible. The inclusion of an economic modelling study, while useful for projecting potential impacts, is a limitation as its findings are based on simulated hypothetical cohorts rather than empirical participant data. Only one study in this review evaluated a direct intervention specifically designed to target suicidal thoughts and behaviours.^28^ Suicide and related thoughts or behaviours were predominantly treated as secondary outcomes of depression or parenting studies, mostly measured via proxies indicators, such as the EPDS Item 10, rather than comprehensive suicide risk assessments such as the Columbia Suicide Severity Rating Scale; and two studies explicitly assessed perinatal suicide as adverse events. Across the four studies evaluating suicidal thoughts via EPDS Item 10, a consistent positive trend emerged. While meta-analysis was not possible due to methodological and clinical heterogeneity, both targeted psychological therapies and structured system-level referral networks showed promise in reducing the proportion of women endorsing self-harm thoughts. Consequently, our findings need to be interpreted with caution even given encouraging results from the task-sharing BA trial. The secondary analysis of the task-sharing with BA trial lacked a non-intervention control arm, limiting our ability to rule out general improvement over time, but the effectiveness of the intervention was established in the parent SUMMIT trial.^46^ Combined with the clear dose–response relationship observed in our pooled data, where each additional session attended was associated with a 25% reduction in the odds of endorsing suicidal ideation, these findings support a treatment effect.

Given the focus within clinical guidelines for managing suicidal thoughts or behaviours on dialectical behavioural approaches, the absence of such approaches in the identified interventions is surprising.^47^, ^48^ Our study complements a recent review of the acceptability and feasibility of interventions for perinatal suicidal behaviour, which primarily focused on pharmacological treatments and excluded non-peer-reviewed studies and those where suicide was a secondary outcome; it also highlighted the importance of whole-family, trauma-informed, integrated care models.^49^ We identified several planned or ongoing trials (Appendix p 35) but future research should prioritise interventions that directly target drivers of suicidal thoughts and behaviours, use validated suicide-specific outcomes, measures self-harm and suicide attempts as well as ideation, and evaluate care models that combine system interventions with multidisciplinary safety nets and access to community support. Given suicide is a rare outcome, target trial emulations and larger-scale population-based approaches might be appropriate to investigate the effectiveness of interventions on perinatal suicide reduction. Further research on informal coping mechanisms, such as faith or community support, are needed to build culturally relevant safety plans for suicide prevention.

In a recent Lancet correspondence, Dandona and colleagues^50^ argued that maternal suicide prevention should adopt a life-course approach, integrating mental health into routine maternal care, and extending support beyond the traditional postpartum period, and improving surveillance to reduce preventable deaths and their consequences for children and families. Echoing this, our review suggests that suicide prevention likely depends on integration of mental health services and coordinated action across multiple levels, combining health and community safety nets for early identification and referral with accessible, evidence-based psychological therapies that address key drivers of suicidal thoughts and behaviours, such as depression and hopelessness. Structured community and system-level interventions and targeted psychological therapies, including tasked-shared BA, look promising. Although the BA evidence is derived from a secondary analysis of a single trial, and its success in this review relies on structured, supervised collaborative care models within well-resourced trials, and its implementation in regions with differing legislative frameworks or scarcer mental health infrastructure requires careful adaptation. Within the broader public health framework,^21^ scalable solutions may lie in embedding prevention within coordinated maternity, mental health, and community systems, strengthening multidisciplinary safety nets and expanding culturally relevant, evidence-based psychological care to ensure that women identified at risk receive timely and effective support. To ensure these safety nets are effective, systems must account for structural barriers and recognise the value of informal community coping mechanisms in bridging gaps to formal care. Ultimately, suicide prevention must be recognised as a core maternal health priority, ensuring that women are supported as whole individuals beyond their parenting roles to improve outcomes for both the mother and the surviving child.

## Contributors

CFT, JS and AE designed the study. CFT, YDE, KDB, AE and JS selected studies for inclusion. CFT, YDE and KDB extracted data, assessed quality and verified the underlying data. All authors (CFT, YDE, KDB, SB, HRJ, CAW, JS and AE) contributed to the analyses and interpretation of the findings. CFT drafted the manuscript and all authors commented and approved the final version of this manuscript.

## Data sharing statement

All data extracted and analysed are included within this article and its Supplementary Information files. No additional original data were generated.

## Declaration of interests

CFT HRJ, KDB, JS and AE declare funding from the NIHR. JS and CFT also declare funding from the FCDO. All other authors declare no competing interests.
