# Supplementary material for: Interventions to prevent suicide and suicidal thoughts or behaviours in the perinatal period: a global mixed-methods systematic review
Source: eClinicalMedicine. 2026 Nov 9;101:104318. doi: 10.1016/j.eclinm.2026.104318 (PMC13770680; doi:10.1016/j.eclinm.2026.104318)
Supplement: Supplementary file 1 [file mmc1.pdf]

Appendix

Search strategy

Keywords			
**Population: (pregnant/parenting women)**	**Intervention Strategy (any)**	**Outcome: Suicide prevention**	
Pregnancy or pregnan*		suicide	Prevention
Parent*		Suicid*	prevent*
mother		Self-harm	reduction
Matern*		Self-injury<br>Self-Injurious Behavior<br>Self-Inflicted Wounds<br>Self-Mutilation	Reduc*
Perinatal or antenatal or postnatal		Overdos*	
		Poison*	

Embase

1. exp high risk pregnancy/ or exp unplanned pregnancy/ or exp unwanted pregnancy/ or exp pregnancy complication/ or exp pregnancy disorder/ or pregnancy.mp. or exp first trimester pregnancy/ or exp adolescent pregnancy/ or exp third trimester pregnancy/ or exp pregnancy/ or exp second trimester pregnancy/
2. exp pregnant woman/
3. exp single parent child/ or exp divorced parent/ or exp parent/ or exp adolescent parent/ or exp expectant parent/ or exp single parent/ or exp separated parent/
4. exp expectant mother/ or exp adolescent mother/ or exp mother/
5. (pregnan* or mother* or parent* or matern*).mp
6. (perinatal or antenatal* or intrapart* or postnatal*).mp.
7. 1 or 2 or 3 or 4 or 5 or 6
8. exp suicide attempt/ or exp suicide/ or exp suicide prevention/
9. exp automutilation/
10. exp intoxication/
11. exp poison/
12. (suicid* or self-harm or self-injury or self-injurious or self-Inflicted or self-mutilation or overdos* or poison*).mp.
13. 8 or 9 or 10 or 11 or 12
14. exp secondary prevention/ or exp prevention/ or exp primary prevention/ or exp suicide prevention/
15. (prevent* or reduc*).mp
16. 14 or 15
17. 7 and 13 and 16
18. limit 17 to (human and female and "remove medline records")

Medline

1. exp Pregnancy Complications/ or exp Pregnancy, Unplanned/ or exp Pregnancy Trimester, Second/ or exp Pregnancy, Unwanted/ or exp Pregnancy Trimester, First/ or exp Pregnancy Trimester, Third/ or exp Pregnancy/ or exp Pregnancy in Adolescence/ or exp Pregnancy Trimesters/ or exp Pregnancy, High-Risk/
2. exp pregnant woman/
3. exp Parents/
4. exp Mothers/
5. exp Maternal Health/
6. exp Prenatal Care/
7. exp Postnatal Care/ or exp Depression, Postpartum/
8. (pregnan* or mother* or parent* or matern*).mp.
9. (perinatal or antenatal* or intrapart* or postnatal*).mp
10. 1 or 2 or 3 or 4 or 5 or 6 or 7 or 8 or 9
11. exp Suicide Prevention/ or exp Suicide/ or exp Suicide, Completed/ or exp Suicide, Attempted/
12. exp Self-Injurious Behavior/
13. exp Self Mutilation/ or exp Wounds, Stab/
14. exp Opiate Overdose/ or exp Drug Overdose/
15. exp Poisons/

16. (suicid* or self-harm or self-injury or self-injurious or self-Inflicted or self-mutilation or overdos* or poison*).mp.
17. 11 or 12 or 13 or 14 or 15 or 16
18. exp Primary Prevention/ or exp Suicide Prevention/ or exp Secondary Prevention/
19. (prevent* or reduc*).mp
20. 18 or 19
21. 10 and 17 and 20
22. limit 21 to (female and humans)

Global Health
1. pregnancy diagnosis/ or pregnancy complications/ or pregnancy/
2. pregnant women/ or pregnant adolescents/
3. parents/
4. mothers/
5. maternity/
6. prenatal care.sh
7. (puerperium or prenatal period).sh.
8. (pregnan* or mother* or parent* or matern*).mp.
9. (perinatal or antenatal* or intrapart* or postnatal*).mp.
10. 1 or 2 or 3 or 4 or 5 or 6 or 7 or 8 or 9
11. suicide/
12. self-harm/
13. wounds.sh.
14. overdose/
15. toxic substances/
16. (suicid* or self-harm or self-injury or self-injurious or self-Inflicted or self-mutilation or overdos* or poison*).mp.
17. 11 or 12 or 13 or 14 or 15 or 16
18. Prevention/
19. (prevent* or reduc*).mp.
20. 18 or 19
21. 10 and 17 and 20

APA PsycInfo
1. exp Pregnancy/ or exp Adolescent Pregnancy/
2. exp Expectant Parents/ or exp Parents/ or exp Homosexual Parents/ or exp Single Parents/
3. exp Parenting/
4. exp Expectant Mothers/ or exp Adolescent Mothers/ or exp Single Mothers/ or exp Mothers/
5. exp Prenatal Care/ or exp Postnatal Period/
6. exp Perinatal Period/
7. (pregnan* or mother* or parent* or matern*).mp.
8. (perinatal or antenatal* or intrapart* or postnatal*).mp
9. 1 or 2 or 3 or 4 or 5 or 6 or 7 or 8
10. exp Youth Suicide/ or exp Attempted Suicide/ or exp Suicide Prevention/ or exp Suicide/ or exp Suicide Prevention Centers/
11. exp Attempted Suicide/ or exp Suicidal Ideation/
12. exp Heroin/ or exp Opiates/ or exp Drug Abuse/ or exp Drug Overdoses/ or exp Intravenous Drug Usage/ or exp Cocaine/ or exp "Opioid Use Disorder"/
13. exp Toxins/
14. (suicid* or self-harm or self-injury or self-injurious or self-Inflicted or self-mutilation or overdos* or poison*).mp.
15. 10 or 11 or 12 or 13 or 14
16. exp "Substance Use Prevention"/ or exp Violence Prevention/ or exp Suicide Prevention/ or exp Suicide Prevention Centers/ or exp Prevention/
17. (prevent* or reduc*).mp.
18. 16 or 17
19. 9 and 15 and 18
20. limit 19 to (human and "remove medline records"

Web of Science
pregnancy OR pregnan* OR mother* OR parent* OR antenatal OR prenatal OR puerperium OR postnatal
AND
suicide OR suicid* OR self-harm OR self-injury OR self-injurious OR self-Inflicted OR self-mutilation or overdos* or poison*
AND
prevent* or reduc*

ASSIA
pregnancy OR pregnan* OR mother* OR parent* OR antenatal OR prenatal OR puerperium OR postnatal
AND
suicide OR suicid* OR self-harm OR self-injury OR self-injurious OR self-Inflicted OR self-mutilation or overdos* or poison*
AND
prevent* or reduc*
Additional filters:
Databases: Psychology Database OR Public Health Database OR Health & Medical Collection OR Research Library OR Research Library: Social Sciences OR Nursing & Allied Health Database OR Science Database OR Sociology Database OR Social Science Database
Documents: all
Languages: all
Remove duplications: Yes

CINAHL
AB (pregnancy OR pregnan* OR mother* OR parent* OR antenatal OR prenatal OR chilbirth OR purperim OR postnatal) AND AB (suicide OR suicid* OR self-harm OR self-injury OR self-injurious OR self-Inflicted OR self-mutilation or overdos* or poison*) AND AB (prevent* or reduc*)
#1. pregnancy OR pregnan* OR mother* OR parent* OR antenatal OR prenatal OR puerperium OR postnatal
#2. suicide OR suicid* OR self-harm OR self-injury OR self-injurious OR self-Inflicted OR self-mutilation or overdos* or poison*
#3. prevent* or reduc*
#4. 1 AND 2 AND 3
Filters: human, exclude Medline records, female

CENTRAL
#1. pregnancy OR pregnan* OR mother* OR parent* OR antenatal OR prenatal OR puerperium OR postnatal
#2. suicide OR suicid* OR self-harm OR self-injury OR self-injurious OR self-Inflicted OR self-mutilation or overdos* or poison*
#3. prevent* or reduc*
#4. 1 AND 2 AND 3

Characteristics of included studies

Study ID: Hassdenteufel 2023	*Reviewers initials: CFT/KDB*

Reference:

Hassdenteufel K, Müller M, Abele H, Brucker SY, Graf J, Zipfel S, Bauer A, Jakubowski P, Pauluschke-Fröhlich J, Wallwiener M, Wallwiener S. Using an electronic mindfulness-based intervention (eMBI) to improve maternal mental health during pregnancy: results from a randomized controlled trial. Psychiatry Research. 2023 Dec 1;330:115599.

Other publications from same study:

Müller, M., Matthies, L.M., Goetz, M., Abele, H., Brucker, S.Y., Bauer, A., et al. 2020. Effectiveness and cost-effectiveness of an electronic mindfulness-based intervention (eMBI) on maternal mental health during pregnancy: the mindmom study protocol for a randomized controlled clinical trial. Trials 21 (1), 933.

Methods	**Study design**: multicenter RCT<br>**Primary aim**: to evaluate the clinical effectiveness of a supervised electronic mindfulness-based intervention (eMBI) in a sample of pregnant women who screened positive for emotional distress<br>**Intervention** (type + pregnancy/postnatal intervention): pregnant and postnatal<br>Baseline measurements: Age, marital status, socio-economic status, education, socio-economic, employment, parity/number of children, pre-existing conditions<br>**Duration of study**: 2019-2021<br>**Ethics**: Approved by Ethics Committees of the Universities of Heidelberg and Tübingen. Registered with German Clinical Trials Registry (DRKS 00,017,210
Participants	Setting: Maternity departments of University Hospitals (Heidelberg and Tübingen) and over 200 gynaecological practices in the state of Baden-Württemberg, Germany<br>**Inclusion criteria**: Women who score >9 on the EPDS (indicating mental distress) and had: age > 18 years; adequate German proficiency; singleton pregnancy; no known neonatal malformations; <29 weeks gestation at screening; statutory health insurance coverage<br>**Exclusion**: Women in acute need of psychiatric treatment (assessed via SCID-5-CV).<br>**Total number of participants**: n= 460 randomized: 230 Intervention; 230 Control (*1153 screened positive for distress out of 5299 total screened)*<br>**Mean age of participants (years) (SD):** study Inclusion (Screening): 21.2 weeks (4.3); Intervention Start (T1)<br>**Mean gestation age at time of intervention start:** provided between weeks 29 and 36. The baseline measurement (T1) was set at the 28th gestational week<br>**OR months after birth at time of intervention start: NA**<br>**Ethnicity** (Country of origin):<br>Germany: Intervention: 109 (87.9%); Control: 140 (83.3%).<br>Other: Intervention: 15 (12.1%); Control: 28 (16.7%)<br>**Marital status**:<br>Married: Intervention: 79 (63.7%); Control: 113 (67.3%),.<br>Partnership: Intervention: 41 (33.1%); Control: 53 (31.5%).<br>Single: Intervention: 4 (3.2%); Control: 1 (0.6%)<br>**Socio-economic status** /**Employment**<br>**< 1500 €:** Intervention: 34 (29.1%); Control: 36 (21.8%).<br>**1500 – 2999 €:** Intervention: 52 (44.4%); Control: 76 (46.1%).<br>**3000 – 4999 €:** Intervention: 23 (19.7%); Control: 39 (23.6%).<br>**> 5000 €:** Intervention: 8 (6.8%); Control: 14 (8.5%<br>Full-time: Intervention: 25 (21.6%); Control: 43 (26.9%).<br>Prohibition notice (medical suspension): Intervention: 45 (38.8%); Control: 68 (42.5%).<br>Part-time: Intervention: 27 (23.3%); Control: 33 (20.6%).<br>Unemployed: Intervention: 19 (16.4%); Control: 16 (10.0%)<br>**Education**:<br>University entrance qualification: Intervention: 52 (42.3%); Control: 97 (58.1%).<br>High secondary qualification: Intervention: 46 (37.4%); Control: 32 (19.2%).<br>University of applied sciences: Intervention: 19 (15.4%); Control: 29 (17.4%).<br>Low secondary/No qualification: Intervention: 6 (4.9%); Control: 9 (5.4%)<br>**Pre-existing medical or mental health conditions:** Current psychotherapy: Intervention: 116 (82.3%); Control: 143 (82.7%).*(All participants screened positive for distress with EPDS >9).*<br>**Other lifestyle/health behaviors**/risk factors<br>No child: Intervention: 62 (50.4%); Control: 105 (62.5%); One child: Intervention: 47 (38.2%); Control: 49 (29.2%); Two or more children: Intervention: 14 (11.4%); Control: 14 (8.3%)

Intervention	**Type and content of intervention** (think Tidier if possible/reported?)<br>*What*: a supervised electronic Mindfulness-Based Intervention (eMBI) specifically developed for this study, titled "mindmom". The program utilized a multidimensional approach combining psychoeducational and obstetrical content, mindfulness exercises such as breathing and body scans, and cognitive behavioural therapy approaches. The content was delivered through audio files, videos, written text, a personal skills box, and interactive worksheets<br>*Who provided*: content was delivered automatically via a smartphone application available on Apple iTunes and the Google Play Store. The intervention was supervised by study staff who monitored submitted data and retained the ability to interact with the patient if necessary<br>*How*: delivered digitally via a smartphone application which participants downloaded to their personal device<br>*Where*: was completed remotely in the participants' own environment using their mobile devices<br>When and how much: Participants were granted access to the application between the 29th and 36th week of pregnancy. The program consisted of eight weekly sessions, with each session lasting approximately 45 minutes.<br>*Tailoring*: the application included a personal skills box and interactive worksheets to facilitate individual engagement. Additionally, the program was supervised, allowing study staff to review data and interact with participants individually if required.<br>*Modifications*: NA<br>*How well (if applicable)*: Adherence was a significant challenge, with 130 randomized participants excluded specifically for noncompliant participation and a 38.26% dropout rate observed in the intervention group. However, the authors described the completion and compliance rates as relatively high compared to previously published online mindfulness trials.<br>**Type and content of control (if applicable):**<br>*What*: Treatment as Usual (TAU), meaning they received standard prenatal care without any study-imposed restrictions or regulations. In addition to standard care, these participants were given access to the study application, but their access was limited to completing bi-weekly digital questionnaires and reading a generic online pregnancy guided book. They did not receive the specific mindfulness, psychoeducation, or CBT content provided to the intervention group.<br>*Who provided*: Standard prenatal care was provided by the participants' regular healthcare providers (e.g., gynaecologists, midwives). The digital component (questionnaires and guided book) was facilitated by the study application.<br>*How*: delivered through routine face-to-face clinical visits.<br>*Where*: typical clinical settings (gynaecological practices and maternity departments).<br>When and how much: Participants received standard care throughout the study period (pregnancy through 5 months postpartum) as per routine schedules. They were required to complete digital questionnaires every two weeks during the 8-week intervention phase and at 1 and 5 months postpartum.<br>*Tailoring*:<br>*Modifications*:<br>*How well (if applicable)*: The control group had a dropout rate of 25.35% (n = 56), which was lower than the intervention group but still notable.
Outcomes	**Outcome considered in the review and reported in the study:**<br>EPDS (Depression); STAI (State-Trait Anxiety); PRAQ-R (Pregnancy-Related Anxiety); FMI-14/FFA-14 (Mindfulness); PHQ-D (Patient Health Questionnaire: stress, somatoform, psychosocial functioning. Baseline (T1) occurred at the 28th Gestational Week<br><br>**Suicidal Ideation (Adverse Event):** Defined as a score >0 on EPDS Item 10.<br>There was no statistically significant difference: 9/142 (6.3%) in the Intervention Group vs. 20/174 (11.5%) in the Control Group. None were judged acutely dangerous.
Underpinning theory of the intervention	Pregnant women face significant barriers to accessing face-to-face mental healthcare, including geographic limitations, financial costs, stigma, etc. Authors think that a supervised digital tool (eMBI) incorporating psychoeducation, mindfulness exercises (e.g., mindful breathing, body scans), and cognitive behavioral therapy (CBT) could help women identify and exit vicious circles of fear regarding birth and parenting. By acquiring these self-regulation skills early in pregnancy (weeks 29–36), women can reduce specific pregnancy-related anxieties and prevent the onset of postpartum depression, thereby optimizing long-term maternal and fetal outcomes.
Challenges & strategies, lessons learn	Issues with adherence and attrition. There was a high dropout rate, particularly in the intervention group (38.26% vs 25.35% in controls), and 130 randomized participants were excluded specifically for "noncompliant participation". To address the lack of personal contact in digital health, the app included a

	supervised element where study staff monitored data and could interact with patients, if necessary, though this was insufficient to prevent high attrition.<br>Authors suggest that while digital interventions are effective for specific targets like birth anxiety, treating general depression may require a "personal component" (face-to-face therapy) that an app cannot fully replace
What is new? (conclusions)	Although the eMBI did not improve general depression or anxiety, it demonstrated a statistically significant reduction in pregnancy-related and birth-related anxiety (PRAQ-R) and significantly improved self-perceived mindfulness scores, The study concludes that eMBI is a valuable, cost-effective tool to complement routine prenatal care for targeting specific pregnancy-related fears, even if it does not replace treatment for general psychopathology
Additional references / Other comments	130 participants (across both groups, though flowchart implies these are separate from dropouts) were excluded for noncompliant participation, though the specific breakdown of compliance within the control group's questionnaire completion is not detailed beyond the final dropout number.

**Quality Assessment – Hassdenteufel 2023**	*Reviewer initials: CFT/KDB*
Critical Appraisal	*The Mixed Methods Appraisal Tool (MMAT) 2018 will be used for quantitative, qualitative and mixed-methods studies.*<br><br>**Screening questions**<br>1. <u>Are there clear research questions?</u> — Yes<br>Yes<br><br>2. <u>Do the collected data allow to address the research questions?</u> — Yes<br>Yes<br><br>**MMAT for quantitative RCT**<br>1. <u>Is randomization appropriately performed?</u> — Yes<br>Randomization list generated by independent assistant using R package "blockrand"<br><br>2. <u>Are the groups comparable at baseline?</u> — Yes<br>Generally yes comparable, but small differences in education and PHQ stress scores were found and controlled for in the analysis.<br><br>3. <u>Are there complete outcome data?</u> — Yes<br>There was substantial dropout (38% in Intervention, 25% in Control). They used Little's MCAR test, but high attrition still affects completeness<br><br>4. <u>Are outcome assessors blinded to the intervention provided?</u> — CT<br>Not possible to blind participants (using an app vs not). Unclear if outcome assessors blinded<br><br>5. <u>Did the participants adhere to the assigned intervention?</u> — CT<br>About 130 participants were marked as "noncompliant participation" in the flowchart

**Study ID: Keefe 2016**	*Reviewers initials: CFT/YDE*
**Reference:**<br>Keefe RH, Brownstein-Evans C & Rouland Polmanteer R. (2016). “I find peace there”: How faith, church, and spirituality help mothers of colour cope with postpartum depression. *Ment Health Relig Cult* 19, 722–733.	
**Other publications from same study:**	

Methods	**Study design**: Qualitative study<br>**Primary aim**: Learn how faith, church participation and spiritual practices of new African American (AA) and Latina mothers who have histories of postpartum depression (PPD) helped them cope with PPD.<br>**Intervention** (type + pregnancy/postnatal intervention): Interviews with new African American and Latina mothers with histories of PPD<br>**Baseline measurements**: Baseline demographic and clinical data were reported for all participants. This included ethnicity, maternal age, history of postpartum depression, current pregnancy status, number of children currently living with the participants, and EPDS scores. The average EPDS (Edinburgh Postnatal Depression Scale) score was 18.75, indicating clinically significant depressive symptoms.<br>**Duration of study**: January 2012 – November 2014<br>**Ethics**: Not reported (funding: Fahs-Beck Fund for Research and Experimentation)
Participants	**Setting**: Large, multi-site, urban-based health centre for low-income residents<br>**Inclusion criteria**: Not directly listed as a criteria however the study mentions that thirty mothers of colour, 19 African American and 11 Latina, were interviewed. They were between the ages of 18 and 44, had histories of PPD, and had between 1 & 7 children living with them. 11 of these were also currently pregnant.<br>**Exclusion criteria**: Not reported.<br>**Total number of participants**: 30 (19 African American, 11 Latina)<br>**Mean age of participants (years) (SD):** not specified, range between 18 and 44 years.<br>**Mean gestation age at time of intervention start: n/a**<br>**OR months after birth at time of intervention start: n/a**<br>**Ethnicity**: African American and Latina<br>**Marital status**: n/a<br>**Socio-economic status**: All participants were recruited from an urban, multi-site health centre serving low-income residents, suggesting the sample consisted of low-income mothers.<br>**Education**: n/a<br>**?Pre-existing medical or mental health conditions:** Post Partum Depression<br>**Other lifestyle/health behaviors/risk factors:** Thoughts of self-harm or suicide (3 mothers)<br>Suicide attempts in lifetime: n/a
Intervention	**Type and content of intervention** (think Tidier if possible/reported?): Faith/church participation<br>*What*: intervention consisted of the mothers' active engagement with their personal faith beliefs, church participation, and various spiritual practices utilized specifically as coping mechanisms for postpartum depression. This involved a combination of internal beliefs (belief in God or a higher power), private practices (praying alone, listening to Christian radio stations, reading spiritual materials), and communal activities (attending church services, being prayed over by others, and confiding in pastors). These practices served as an informal support system that mothers used to manage symptoms such as sadness, fatigue, etc.<br><br>*Who provided*: Support was provided primarily by the faith community and the church structure. The providers included fellow church members who "gathered around" the mothers to offer emotional support, making them feel valued and less alone, and pastors who served as confidants for mothers struggling with depression. Additionally, the mothers viewed God or a "Higher Power" as a direct provider of guidance, strength, and peace. The mothers themselves also acted as providers of mutual aid, using their faith to develop empathy and offer practical support (such as help finding housing) and prayer for other women in similar situations.<br><br>*How*: The intervention functioned through specific spiritual and cognitive mechanisms that relieved stress and shifted the mothers' perspectives on their suffering. Prayer operated as an immediate coping strategy for acute distress; mothers described "talking to Him out loud" to release sadness and noted that prayer was a critical protective factor against self-harm, with one mother explicitly stating she did not die by suicide because she believed she "could not ask God for forgiveness" if she hurt herself. Church attendance provided a mechanism for emotional regulation, offering a sense of peace and calmness that allowed mothers to "let go" of worries and feel important when blessed by others. Furthermore, believing in God's

	guidance empowered mothers to leave negative relationships and take responsibility for changing their lives, attributing their renewed strength and energy to this spiritual connection.<br>*Where*: The intervention took place across multiple settings: explicitly within the church building, which mothers described as a sanctuary where they found peace and happiness on Sundays, and within their private homes and daily environments. Private spiritual practices were integrated into daily life outside the church, such as listening to Christian radio stations at home or praying alone during moments of overwhelming stress/suicidal ideation.<br>When and how much: Engagement with the intervention was frequent, self-directed, and ongoing throughout the perinatal period. While church attendance typically occurred weekly on Sundays, prayer was used as a daily practice. One mother reported that during her lowest points, there was "never a day where that praying did not work," and another described praying "the majority of the time" when facing the urge to give up or harm herself<br>*Tailoring*: *Modifications*: Tailored to the cultural and social realities of the African American and Latina mothers interviewed; the practices were highly personalized to each mother's specific context, e.g. mothers adapted their spiritual focus to address their unique stressors, such as housing instability, relationship conflicts with the baby's father, or the need to bond with their infants. The study notes that this culturally relevant support system succeeded in meeting needs where formal services often failed, by validating the mothers' lived experiences and offering a sense of community belonging.<br>*How well (if applicable)*: n/a<br><br>**Type and content of control (if applicable): n/a**
	**Outcome considered in the review and reported in the study:**<br>**Preventing self-harm**<br>One mother's spiritual beliefs helped prevent her from harming herself when she was at a very low emotional point. Other mothers' spiritual beliefs led to faith practices that prevented them from self-harm, such as praying. Specific mechanisms mentioned include:<br>- Protection: One mother noted that suicide was contrary to God's will: "*I know if I was to hurt myself I could not ask God for forgiveness*".<br>- Immediate symptom relief: Another mother described prayer as an acute intervention for suicidal ideation: "*There was never a day where that praying did not work... all the sudden your spirit just lift up*".<br>- Frequency: The study explicitly states that three mothers identified faith as the specific factor that prevented them from acting on suicidal thoughts<br>**Other outcomes:**<br>Relief from stress: Prayer had a lot of power for these women, and they felt as though the act of praying provided a listening ear and relieving presence from their stress and overwhelm. There is a recurring theme of peace and stress relief in prayer and in attending church that helped release stress from life.<br>Feeling valued and less alone: Attending church and regularly communing with fellow church members allowed women to feel seen and valued by others. The community being able to pray for and over them made them feel incredibly supported, specially emotionally.<br>Experiencing gratitude: Having faith in a higher power allowed these women to find not only peace but gratitude in their sufferings. Whilst motherhood was hard, they were able to see the good things that came from motherhood. In addition, attending church allowed the building of connections with others and opened opportunities for the development of new perspectives and growing in relationships, developing empathy for others and preventing self-harm.<br>Developing perspective and accepting God's guidance: After praying, many mothers described how God showed them the way toward change: they only needed to be open to receiving God's guidance. While giving credit to God, many of the mothers also acknowledged their part by being open to God's guidance and using the strength they derived from their prayers, faith, and relationship with God to take action.<br>Changing and developing relationships: The mothers described changes in their relationships including being more open and connected with family members, surrounding themselves with positive people, sharing their experiences, engaging in mutual aid, and developing empathy. They attributed the changes to their ongoing spiritual beliefs and practices. These mothers were able to enjoy spending time with friends again and being surrounded by positivity helped them feel empowered.
Underpinning theory of the intervention	The study is grounded in a biopsychosocial/spiritual perspective, acknowledging that for African American and Latina mothers, spiritual beliefs are not merely religious observances but active coping mechanisms for mental health challenges like PPD. The theory suggests a pathway of change where specific practices (prayer, church attendance) lead to immediate emotional shifts, such as relief from stress, a sense of peace, and feeling value, which then facilitate cognitive restructuring. By surrendering worries to a "Higher Power" and accepting God's guidance, mothers can develop new perspectives on their stressors, leading to tangible behavioural changes such as improved family relationships and the prevention of self-harm.

Challenges & strategies, lessons learn	Authors are White, middle-class professors interviewing low-income Latin American and Black mothers, which the authors acknowledged might have limited the depth of experiences the mothers were willing to share, alongside researchers' initial lack of specific knowledge regarding the role of faith in these communities. To mitigate these barriers, the team employed strategies e.g. actively exploring local churches, consulting with faith leaders and agency staff and maintaining ongoing consultation with experts in minority health research. The study highlights that because these mothers often encounter prejudice in formal healthcare settings, they frequently rely on informal support systems like the church.<br>Lessons include the need mental health practitioners to integrate a mother's spiritual perspective into clinical assessments and to foster formal collaborations between clinicians and faith-based organizations, such as hosting support groups within churches, to provide culturally relevant, non-stigmatizing care that prevents the social isolation often associated with postnatal depression.
What is new? (conclusions)	This study adds to the growing body of literature on the effects of church participation and spirituality on health and well-being and helps to advance the relevance of the Biopsychosocial/Spiritual perspective to mothers with postnatal depression. It provides new evidence that faith practices serve as specific protective factors against PPD symptoms, identifying distinct mechanisms such as "preventing self-harm" through theological deterrence (believing suicide is contrary to God's will) and the use of prayer as an immediate intervention for acute distress
Additional references / Other comments	

**Quality Assessment – Keefe 2016**	*Reviewer initials: CFR/YDE*
Critical Appraisal	*The Mixed Methods Appraisal Tool 2018 will be used for quantitative, qualitative and mixed-methods studies.*<br><br>**Screening questions**<br>1. <u>Are there clear research questions?</u><br>How faith, church participation and spiritual practices helped African American and Latina mothers cope with postpartum depression (PPD) — Yes<br><br>2. <u>Do the collected data allow to address the research questions?</u> — Yes<br>Data collected via 30 semi-structured, face-to-face interviews with the specific target population are appropriate to explore these lived experiences<br><br>**MMAT for qualitative studies**<br>1. <u>Is the qualitative approach appropriate to answer the research question?</u><br>Qualitative approach is suitable for exploring the subjective processes ("how") and coping strategies of mothers, which cannot be fully captured by quantitative measures — Yes<br><br>2. <u>Are the qualitative data collection methods adequate to address the research question?</u><br>Use of semi-structured interviews allowed for depth and flexibility. The interview guide was refined through pilot testing with 12 RAs, and data were digitally recorded and transcribe — Yes<br><br>3. <u>Are the findings adequately derived from the data?</u><br>Yes – themes were derived and supported by patient answers. The themes were clearly linked to the interview content (rigorous line-by-line coding, thematic coding. comparative analysis). Coding independently by three authors. — Yes<br><br>4. <u>Is the interpretation of results sufficiently substantiated by data?</u> — Yes<br>Six identified themes well supported by quotes from participants (e.g., specific quotes on prayer preventing self-harm)<br><br>5. <u>Is there coherence between qualitative data sources, collection, analysis and interpretation?</u> — Yes<br>There is strong coherence between the biopsychosocial/spiritual theoretical framework, interview data collection method and thematic analysis, and conclusions regarding clinical practice.

**Study ID: Liepman 2011**	*Reviewers initials: CFT/YDE*

Reference:

Liepman MR, Kothari CL, Tareen RS, Florian PM, Charoth RM, Sweezy LC, Haas SS & McKean JW. (2011). Community-wide intervention reduces postpartum depression through promotion of perinatal screening and treatment. *Marce International Society Biennial Meeting Abstract Book.*

Other publications from same study:

Methods	**Study design**: Repeated cross-sectional (quasi-experimental).<br>**Primary aim**: To evaluate a community-wide educational and advocacy intervention to improve care for women with perinatal mood disorders by increasing/standardizing earlier screening, improving referral/acceptance, and enhancing community awareness.<br>**Intervention** (type + pregnancy/postnatal intervention): community-wide intervention (training, advocacy, clinical services) to promote perinatal screening and treatment.<br>**Baseline measurements**: 2002 cohort data serves as the baseline comparison<br>**Duration of study**: 2002 – 2009 (baseline data collection: 2002; Intervention period: 2007-2009; post-intervention data collection: 2009)<br>**Ethics**: Not reported in abstract.
Participants	**Setting**: Kalamazoo, Michigan, USA<br>**Inclusion criteria**: Hospitalised postpartum women. No other inclusion/exclusion criteria mentioned<br>**Total number of participants**: Two random 10% samples of hospitalized postpartum women were enrolled = 658 total (332 in 2002 cohort; 326 in 2009 cohort).<br>**Mean age of participants (years) (SD) [min, max]:** Not reported.<br>**Mean gestation age at time of intervention start:** Not reported.<br>**OR months after birth at time of intervention start:** Not reported**.**<br>**Ethnicity**: Not reported<br>**Marital status**: Not reported<br>**Socio-economic status**: Not reported<br>**Education**: Not reported<br>**Pre-existing medical or mental health conditions:** Not reported<br>**Other lifestyle/health behaviors:** 'Intimate partner abuse and stress" measured as predictors.
Intervention	**Type and content of intervention** (Tidier if possible/reported): training healthcare and social service providers alongside public service announcements on PMH<br>*What*: The core of the program involved training nearly 500 area healthcare and social service providers on critical topics including epidemiology, screening protocols, treatment options, referral pathways, and the safety of various psychotropic drugs during pregnancy and lactation. Alongside this professional development, the project established a specialized Women's Behavioral Health Clinic designed to evaluate complex cases, offer consultative advice to prenatal providers, and train future psychiatrists. Additionally, the intervention involved the compilation and distribution of tangible resources, such as lists of interested mental health providers and support groups, as well as the widespread printing and distribution of educational literature regarding perinatal mood disorders<br>*Who provided*: The initiative was conducted through a collaboration between Michigan State University/Kalamazoo Center for Medical Studies, Healthy Babies Healthy Start, and Western Michigan University. On the ground, health promoters and case managers were responsible for disseminating the public health messages to the wider community, while the Women's Behavioral Health Clinic provided specialist training for future psychiatrists<br>*How*? The intervention utilized a multi-modal delivery approach combining direct professional education with mass media strategies. This included the broadcasting of public service announcements about perinatal mood disorders on local radio stations to reach the general public, while the clinical component provided expert consultation and direct patient evaluation for high-risk cases<br>*Where*: Implemented across the wider community of Kalamazoo, Michigan, engaging local healthcare and social service networks.<br>*When and how much:* The program was delivered over an 18-month period between 2007 and 2009. While the specific frequency of literature distribution, radio station announcements and Women's Behavioral Health clinic activity were not reported., the training component reached nearly 500 providers during this timeframe.

	Tailoring: not mentioned<br>*Modifications*: not mentioned<br>*How well (if applicable)*: There was no mention of planned and actual results of the intervention. There was also no assessment of fidelity to the assessment plan.<br><br>**Type and content of control (if applicable, Tidier if possible/reported)**: Care as usual. Comparison group consisted of 332 hospitalized postpartum women recruited in 2002 using the same random sampling methodology (a random 10% sample) as the intervention group. The 2002 cohort represents the standard level of community care and provider behaviour regarding perinatal mood disorders prior to the implementation of the training, advocacy, and clinical services which began in 2007. The effectiveness of the intervention was established by contrasting the low screening and high depression rates in this 2002 baseline group against the significantly improved metrics observed in 2009.
Outcomes	**Outcome considered in the review and reported in the study:**<br>The average EPDS depression score for the population dropped significantly from 7.53 to 5.08. Regarding suicidality, the frequency of women answering "yes" to the question on having suicidal thoughts was significantly reduced, dropping from 7.1% in the baseline group to 3.5% in the post-intervention group ($p<0.001$)<br>Other: The study reported statistically significant improvements across all process and mental health measures. Regarding screening and diagnosis, documented screening in prenatal records increased nearly nine-fold from 10.6% in 2002 to 91.4% in 2009, while provider identification of depression rose from 2.4% to 14.7%. Treatment rates also more than tripled during this period.
Underpinning theory of the intervention	Whilst there was no theory mentioned, the intervention seems to be based on the assumption that community-wide education and advocacy lead to increased provider screening and referrals, which increases the uptake of professional help, ultimately reducing depression and suicidality.
Challenges & strategies, lessons learn	Challenges not reported in abstract. The study demonstrates that reporting professional help is key mechanism (strongest predictor) for reducing depression, validating the strategy of increasing access/referrals.
What is new? (conclusions)	A community-wide educational and advocacy intervention, accompanied by specific treatment support (referral pathways/specialist clinic), can successfully alter healthcare provider behaviours to raise the standard of care. This systemic change significantly reduces both postpartum depression symptoms and suicidality levels in the community.
Additional references / Other comments	Only abstract, so it was difficult to extract information – authors contacted several times but no response.

**Quality Assessment – Liepman 2011**	*Reviewer initials: CFT/YDE*
Critical Appraisal	*The Mixed Methods Appraisal Tool (MMAT) 2018 will be used for quantitative, qualitative and mixed-methods studies.*<br><br>**Screening questions**<br>1. <u>Are there clear research questions?</u> — Yes<br>While the abstract does not state a formal research question, it provides a clear objective: to reduce rates of perinatal depression through community-wide interventions involving education, screening, and access to care.<br><br>2. <u>Do the collected data allow to address the research questions?</u> — Yes<br>Changes in screening, diagnosis, treatment, and EPDS scores seen. However, as only the abstract was available, unable to assess depth, completeness, or rigour of the data collection methods.

	**MMAT for quantitative non-randomised studies**<br>1. Are the participants representative of the target population? — Yes<br>Robust recruitment method by enrolling two random 10% samples of hospitalized postpartum women. As most births occur in hospital settings, representative sample<br><br>2. Are measurements appropriate regarding both the outcome and intervention (or exposure)? — Yes<br>Used EPDS tool for measuring depression and suicidality, alongside objective medical record abstraction for screening rates.<br><br>3. Are there complete outcome data?<br>Abstract does not provide a flow chart or attrition — CT<br><br>4. Are the confounders accounted for in the design and analysis?<br>Authors used linear modeling for statistical analysis and mentions that they analysed potential confounders and predictors such as intimate partner abuse and stress, but not sure if others missed — CT<br><br>5. During the study period, is the intervention administered (or exposure occurred) as intended?<br>Probably. Abstract describes a successful rollout over 18 months, including the training of nearly 500 providers and the successful establishment of the clinical service. — Yes

**Study ID: Ragesh 2020**	*Reviewers initials: CFT/KDB*
**Reference:**<br>Ragesh, G., et al. "Feasibility, acceptability and usage patterns of a 24-hour mobile phone helpline service for women discharged from a mother-baby psychiatry unit (MBU) in India." Indian journal of psychological medicine 42.6 (2020): 530-534.	
**Other publications from same study:**	

Methods	**Study design**: exploratory, cross-sectional study<br>**Primary aim**: to assess the feasibility, acceptability, limitations, and usage patterns of a helpline service for assess the feasibility, acceptability, limitations, usage patterns, and users characteristics of a free 24-hour mobile phone helpline for mothers and families discharged from an mother and baby unit.<br>**Intervention** (type + pregnancy/postnatal intervention): a24-Hour Mobile Phone Helpline Service<br>**Baseline measurements**: Maternal age, infant age, diagnosis, education, socio-economic status, location, urban/rural setting.<br>**Duration of study**: 2015-2017
Participants	**Setting**: Mother and Baby Unit at National Institute of Mental Health and Neurosciences, Bangalore, India. MBU is a five-bedded inpatient unit where mothers are admitted with their infants and one caregiver. Multidisciplinary team. Predischarge counselling including information regarding handling crises and attending follow-up appointments. Follow up at perinatal outpatient service until infant is two years old.<br>**Inclusion criteria**: All mothers (pregnant and postpartum) admitted from June 2015 to December 2016 (18 months). Contacted six months after their discharge.<br>**Total number of participants**: 113<br>**Mean age of participants (years) (SD):** callers: 25.23 ± 4.14; non-callers: 24.19 ± 4.92<br>**Mean gestation age at time of intervention start: n/a**<br>**OR months after birth at time of intervention start:** n/a<br>**Ethnicity**: Not collected.<br>**Marital status**: n/a<br>**Socio-economic status**:<br>Above poverty line: 20(39.2%); non-caller: 32(51.6%)<br>Below poverty line: 30(58.8%); non-caller: 30(48.4%)<br>Urban: 21(41.2%); non-caller: 22(35.5%)<br>Rural: 30(58.8%); non-caller: 40(64.5%)<br>**Education**:<br>Primary level education: 21(41.2%); non-caller: 27(43.5%)

	Secondary level education or more: 20(39.2%); non-caller: 27(43.5%)<br>**Pre-existing medical or mental health conditions:**<br>Acute and transient Psychotic disorder: caller: 12(23.5%) ; non-caller: 24(38.7%)<br>Depression: caller: 14(27.5%); non-caller: 9(14.5%)<br>Bipolar disorder: caller: 10(19.6%) ; non-caller: 13(21%)<br>Others: caller: 15(29.4%) ; non-caller: 16(25.8%)<br>**Other lifestyle/health behaviors**/risk factors? n/a
Intervention	**Type and content of intervention** (think Tidier if possible/reported)<br>*What*: a 24-Hour Mobile Phone Helpline Service for Women Discharged from a Mother-Baby Psychiatry Unit.<br>*Who provided*: The phone was held by the psychiatric social worker of the perinatal psychiatry team. The psychiatric social worker in the MBU team is part of all clinical discussions and participates in perinatal mental health teaching and training as well. The reason for assigning this task to the psychiatric social worker was related to the prior experience that most of the calls received from this population through the hospital telephone switchboard were for psychosocial support. If the psychiatric social worker was on leave, another team member took over the task.<br>An algorithm developed by the multidisciplinary team was used for handling the call, and other team members were contacted in case of need.<br>*How*: During predischarge counselling, all mothers (both pregnant and postpartum) were given the helpline number in a printed card. The mother and her relatives present at the time of predischarge counselling were provided information about how and when to use the helpline. They were encouraged to use the service during working hours (9:00 am to 5:00 pm) as far as possible, unless it was an emergency.<br>*Where*: MBU, Mobile Phone Helpline<br>When and how much: Pregnancy and postnatal periods.<br>The algorithm included standard responses for possible questions and SOPs for handling crises such as risk to the mother or the baby, an exacerbation of symptoms, or a risk of suicidality. Medical queries were discussed with the psychiatrist in the team. In case of self-harm or other health risk, mothers or their family members were advised to contact the local psychiatric emergency services or reach our hospital psychiatric emergency service. Mothers who reported DV and required shelter and safety were either advised to come to the perinatal psychiatry clinic and/or appropriate services and referrals were provided to other agencies. Those who did not have any acute or serious issues related to health were provided advice over the phone or asked to come for an early follow-up at the perinatal psychiatry clinic.<br>*Tailoring*: not reported.<br>*Modifications*: n/a<br>*How well (if applicable)*: n/a<br><br>**Type and content of control (if applicable): NA**
Outcomes	**Outcome considered in the review and reported in the study:**<br><br>**Reasons for Using the Helpline:**<br>*Calls related to mother:*<br>Reporting exacerbation in symptoms and seeking help to get inpatient care: 21<br>Help regarding psychiatry emergency services for mother: 8<br>Advice regarding dosage and side effects of medications: 11<br>Advice regarding sleep problems: 5<br>**Help to deal with suicidal ideation: 2**<br><br>Calls related to the baby<br>Breastfeeding-related doubts: 9<br>Asking for fetal anomaly scanning services in pregnant mothers: 4<br>Issues related to child custody: 1<br>Health problems in the infant: 1<br><br>Calls related to social and partner-related issues<br>Discussing interpersonal problems: 18<br>Reporting crisis and request for shelter: 13<br>Discussing poor support from spouse and family: 6<br>Reporting domestic violence: 6<br>Help for substance use in the spouse: 1<br>Appointment related and other issues<br>Outpatient appointment: 134

	Calls from surgeons/gynaecologists/physicians through patients or family members regarding contraception and use of no psychotropic medications: 5<br>Referrals to local psychiatrists: 3<br><br>**Other: acceptability / usefulness**<br>• 76 of 113 mothers (67.2%) followed up<br>• Of the 76, 57.9% (44) had used the phone helpline. 50% (n = 22) of the calls were made by spouses, 40.9% (n = 18) of the calls were by the women, and a minority by relatives.<br>• Of the 76 contactable mothers: nearly half had not made any calls (32).<br>• Among the 32 non-callers, 72% (23) knew that there was a telephone helpline available. Reasons for not contacting included not facing any illness-related problem (68.75%) or having contacted a local doctor for the same. However, 25 (8) reported not knowing the helpline number.<br>• Of the 32 non-callers contacted, one phone number belonged to the brother of a woman who had died by suicide and had not contacted the service for help. He mentioned that the helpline phone number was with him and that the patient had not owned a cell phone.<br>• Of the 44 users, 93% (41) said that it was a useful service, 91% (40) said they got a helpful response, 95% (42) said they would recommend this service to others, 9% (4) reported language and communication difficulties, and 5% (2) reported problems in getting in touch.<br>• The average rating of the service was 8.96 (minimum 7, maximum 10).
Underpinning theory of the intervention	No explicit ToC/logic model but there is an implicit rationale that mobile technology can bridge the service gap in LMIC countries where women with perinatal mental illness face significant barriers to accessing physical care. Authors states that a helpline facilitates collaborative care by connecting women directly to a multidisciplinary team for symptom management, health education, and crisis intervention, effectively overcoming infrastructure limitations and empowering women to seek help independently despite potential gender disparities in technology access.
Challenges & strategies, lessons learn	Main challenges included communication barriers due to language differences, difficulties in managing complex interpersonal problems or persuading families to seek inpatient care during crises (such as suicidal risk), and logistical issues where women lacked direct phone access (e.g. case where a woman died by suicide while her brother held the helpline number). To mitigate this, a psychiatric social worker supported by a multidisciplinary team and SOPs to manage medical queries and emergencies effectively.<br>Key lessons learned were that mobile helplines are highly feasible and empower women to seek collaborative care independently despite gender gaps in phone ownership; however, future services must ensure the primary caregiver receives the contact information and establish specific crisis plans for women who do not own a mobile phone.
What is new? (conclusions)	Following discharge from MBU, a telephonic access to a dedicated multidisciplinary team that practices collaborative care and promptly handles the aforementioned needs is necessary. The<br>helpline users reported a positive experience of using the service and mentioned that they would recommend the service to others with similar issues. A dedicated team member could handle the phone with support from the multidisciplinary team, without feeling unduly burdened. The helpline appears to be feasible, acceptable, and useful in most instances and can be used in other LMIC settings.
Additional references / Other comments	*Still not evidence it reduces suicidality or not - needed before scaling up in India or other LMIC.

**Quality Assessment – Ragesh 2020**		
Critical Appraisal	*The Mixed Methods Appraisal Tool (MMAT) 2018 will be used for quantitative, qualitative and mixed-methods studies.*<br><br>**Screening questions**<br>1. <u>Are there clear research questions?</u><br>The study explicitly states the aim is to assess the feasibility, acceptability, limitations, usage patterns, and users characteristics of a free 24-hour mobile phone helpline.	Yes

	2. Do the collected data allow to address the research questions?<br>Authors collected objective call logs for usage patterns and conducted follow-up to assess acceptability and reasons for non-use, which directly answers the research questions.	Yes
	**MMAT for quantitative descriptive**<br>1. Is the sampling strategy relevant to address the research question?<br>The study targeted population of interest: all mothers discharged from the Mother-Baby Unit (MBU) over an 18-month period (census sampling).	Yes
	2. Is the sample representative of the target population?<br>They attempted to recruit the entire cohort of discharged mothers (n=113). They also analysed socio-demographics to confirm no significant differences between those who used the service and those who did not (Table 2).	Yes
	3. Are the measurements appropriate?<br>Usage data was based on objective call logs (not recall). Acceptability was based on direct interviews with users and non-users 6 months post-discharge.	Yes
	4. Is the risk of nonresponse bias low?<br>While they attempted to contact everyone, they only reached 76/113 mothers (67.2%). 33% were lost to follow-up, which could introduce some risk of bias	No
	5. Is the statistical analysis appropriate to answer the research question?<br>Descriptive statistics (frequencies, %) which might be appropriate for feasibility study. Chi-square and T-tests used to compare characteristics of callers and non-callers.	Yes

Study ID: Rosan 2025 *Reviewers initials: CFT/KDK*

Reference:

Rosan C, Alyousefi-van Dijk K, Cornelius V, et al. Clinical effectiveness of the Circle of Security-Parenting group intervention for birthing parents in perinatal mental health services in England (COSI): a pragmatic, multicentre, assessor-masked, randomised controlled trial. *Lancet Psychiatry*. 2025; 12: 817–29.

Other publications from same study:

Rosan C, Dijk KA, Darwin Z, et al. The COSI trial: a study protocol for a multi-centre, randomised controlled trial to explore the clinical and cost-effectiveness of the Circle of Security-Parenting Intervention in community perinatal mental health services in England. Trials 2023; 24: 188.

Methods	**Study design**: Pragmatic, multicentre, randomised controlled trial with an internal pilot.<br>**Primary aim**: to evaluate whether the hybrid delivery of Circle of Security-Parenting (COS-P) plus treatment-as-usual reduces psychopathology in birthing parents accessing NHS community perinatal mental health services, compared with treatment-as-usual alone.<br>**Intervention** (type + pregnancy/postnatal intervention): postnatal (participants were birthing parents of a child aged <1 year).<br>**Baseline measurements**: CORE-OM (psychopathology); PBQ (bonding); DERS (emotion regulation); demographic data (age, ethnicity, education, living situation); child info (age, sex); mental health history.<br>**Duration of study**: 2022-2025<br>**Ethics**: NHS Surrey Research Ethics Committee (21/LO/0723). Registered as ISRCTN18308962.
Participants	**Setting**:10 NHS Trusts across England (Cheshire, Merseyside, Yorkshire, Cumbria, Northamptonshire, Devon, Sussex, Hampshire) with existing specialist community perinatal mental health services.<br>**Inclusion criteria**: aged 18 years; receiving care from participating community perinatal mental health service; clinical-level psychopathology (average CORE–OM 1.1); bonding difficulties (total PBQ 12); birthing parent of a child <1 year.<br>**Exclusion**: currently experiencing active psychosis or previously received COS-P.<br>**Total number of participants**: 371 (248 intervention; 123 control) (3171 screened).

	**Mean age of participants (years) (SD):** intervention: 30.7; control: 31.1<br>**Mean gestation age at time of intervention start:** NA. Child age at baseline: 21.4 weeks (12.6).<br>**OR months after birth at time of intervention start:**<br>**Ethnicity**:<br>White (British, Irish, Other): intervention: 216 (87%); control: 113 (92%).<br>Mixed: intervention: 6 (2%); control: 3 (2%).<br>Asian or Asian British: intervention: 2 (1%); control: 1 (1%).<br>Black or Black British: intervention: 1 (<1%); control: 0 (0%).<br>Other ethnic group: intervention: 1 (<1%); control: 1 (1%).<br>**Marital status**:<br>Living with partner: intervention: 188 (76%); control: 96 (78%).<br>Living alone: intervention: 21 (8%); control: 10 (8%).<br>Living with other relatives: intervention: 13 (5%); control: 11 (9%).<br>Living with others: intervention: 4 (2%); control: 1 (1%).<br>Missing/Prefer not to say: intervention: 22 (9%); control: 5 (4%).<br>**Socio-economic status**: Not reported.<br>**Education**:<br>Higher education: intervention: 127 (51%); control: 70 (57%).<br>Tertiary or further education: intervention: 75 (30%); control: 36 (29%).<br>Secondary: intervention: 17 (7%); control: 9 (7%).<br>Primary or less: intervention: 4 (2%); control: 0 (0%).<br>Other: intervention: 2 (1%); control: 2 (2%).<br>**Pre-existing medical or mental health conditions:**<br>Depression: intervention: 204 (82%); control: 97 (79%).<br>Anxiety: intervention: 199 (80%); control: 101 (82%).<br>Trauma: intervention: 101 (41%); control: 42 (34%).<br>Personality difficulties: intervention: 36 (15%); control: 20 (16%).<br>Obsessive Compulsive Disorder (OCD): intervention: 32 (13%); control: 11 (9%).<br>Bipolar disorder: intervention: 10 (4%); control: 5 (4%).<br>Psychosis: intervention: 10 (4%); control: 2 (2%)<br>**Other lifestyle/health behaviors**/risk factors. Not reported
Intervention	**Type and content of intervention** (think Tidier if possible/reported): COS-P<br>*What*: Circle of Security-Parenting (COS-P), a transdiagnostic, attachment-informed group programme that integrates psychoeducational, cognitive–behavioural, and psychodynamic theories. The curriculum consists of 8 modules covering key topics such as foundational attachment principles, responding effectively to children's emotional states, addressing parenting difficulties, and recognising hostile, helpless, or neglectful caregiving behaviours. The sessions used video examples of parent–child interactions and reflections from previous participants to facilitate discussions about the parents' own attachment and parenting experiences.<br>*Who provided*: Psychological practitioners, including clinical psychologists, cognitive behavioural therapists, or parent–infant psychotherapists. While these practitioners had previous experience in facilitating psychological group work, they were newly trained in COS-P specifically for the trial. Their training was provided by COS-International (intervention developers) and consisted of a standard 24-hour online course, a 1.5-hour workshop focused on perinatal adaptations, and 20 hours of coaching supervision.<br>*How*: delivered in a group format using a hybrid model. Sessions involved viewing video materials and engaging in reflective group discussions facilitated by the trained interveners.<br>*Where*: delivery was split between physical and digital settings. Where possible, two sessions (the initial session and one additional session) were conducted face-to-face in group rooms at NHS Trusts or accessible local venues such as libraries (remaining sessions were online via video call)<br>When and how much: ten weekly sessions, with each session lasting 90 minutes. Participants were generally expected to start the intervention within four weeks of randomisation<br>*Tailoring*: A specific perinatally adapted manual was developed by the programme's developers for use in this trial, differing from the standard COS-P manual designed for children aged 4 months to 6 years. This adaptation was necessary to address the needs of birthing parents with infants younger than one year.<br>*Modifications*: Not reported.<br>*How well (if applicable)*: Adherence variable, participants attending a mean of 6.4 out of 10 sessions (median of 8). Intervention fidelity was monitored by independent raters using video recordings of group sessions. In total, 51 COS-P groups were delivered by 21 different interveners.<br><br>**Type and content of control (if applicable):**<br>*What*: Treatment-as-usual (TAU), which remained unchanged by participation in the trial. This care followed national service specifications and consisted of multidisciplinary, needs-based care tailored to the individual. Services provided could include psychiatric reviews, pharmacological interventions, care coordination,

	parent–infant relational interventions (e.g., baby massage), psychological therapies (e.g., cognitive behavioural therapy), and psychosocial support such as occupational health services or peer support<br>*<u>Who provided</u>*: standard clinical staff within the participating NHS specialist mental health services<br>*<u>How</u>*: routine clinical practice, which typically involves face-to-face clinical appointments, home visits, or other standard delivery methods used by the service.<br>*<u>Where</u>*: NHS community perinatal mental health services<br><u>When and how much</u>: Participants received standard care throughout the duration of the study (up to the 12-month follow-up) according to clinical needs and routine care<br>*<u>Tailoring</u>*: TAU is defined as "needs-based care," meaning the specific combination of therapies (medication, CBT, peer support) was determined by the clinical requirements of each patient<br>*<u>Modifications</u>*: Not reported.<br>*<u>How well (if applicable)</u>*: Not specifically reported. Follow-ups were 75% at 3 months, dropping to 63% at 12 months. In terms of safety, 17% of participants in the control group reported adverse events, compared to 21% in the intervention group.
Outcomes	**Outcome considered in the review and reported in the study:**<br>**Suicidality related: self-harm (as adverse event)**<br>Self-harm or concerns about self-harm reported by 11 participants (3%) total (9 in Intervention vs 2 in Control)<br>Serious Adverse Events (SAE): 2 participants (1%) reported SAEs related to self-harm/concerns about self-harm. 7 participants (2%) reported SAEs of increased mental health difficulties requiring inpatient stay.<br>Primary: Psychopathology (CORE–OM) averaged across 3, 7, and 12 months.<br>Secondary: Postpartum bonding (PBQ); emotion regulation (DERS); child Development (ASQ3, ASQ-SE); maternal sensitivity (NICHD scales); infant attachment (Strange Situation Procedure SSP).
Underpinning theory of the intervention	COS-P is based on the premise that perinatal mental health difficulties often compromise the parent–infant relationship. COS-P is an attachment-informed, transdiagnostic intervention designed to target parental emotion regulation and "reflective functioning" (ability to understand the infant's needs). By strengthening these capacities within a supportive group environment, the intervention aims to improve parent–infant bonding, which is theorized to subsequently reduce parental psychopathology and optimize long-term child developmental outcomes.
Challenges & strategies, lessons learn	Adherence was variable (mean 6.4 sessions) and applying attachment concepts to newborns proved "abstract" for parents. Additionally, high levels of emotion regulation difficulties persisted in both groups, suggesting the 10-week curriculum was insufficient for this population's severity.: A specific perinatally adapted manual was developed, and a hybrid delivery model (combining face-to-face and online sessions) was used to balance clinical engagement with practical constraints. Authors conclude that a standard parenting group is likely insufficient for parents with complex perinatal mental health needs. Future strategies should adopt "precision-based" approaches, potentially reserving such interventions for specific subgroups (e.g., those with personality difficulties) or using more intensive therapeutics
What is new? (conclusions)	COS-P + TAU did not demonstrate greater clinical effectiveness compared to TAU alone. There were no significant differences in psychopathology, bonding, or child outcomes. COS-P is not recommended for inclusion in routine community perinatal mental health services for this population.
Additional references / Other comments	

**Quality Assessment – Rosan 2025**	*Reviewer initials: CFT/KDB*
Critical Appraisal	*The Mixed Methods Appraisal Tool (MMAT) 2018 will be used for quantitative, qualitative and mixed-methods studies.*<br><br>**Screening questions**<br>1. <u>Are there clear research questions?</u> Yes<br>To evaluate effectiveness of of Circle of Security-Parenting (COS-P) plus treatment-as-usual reduces psychopathology in birthing parents accessing NHS community perinatal mental health services

	2. Do the collected data allow to address the research questions?<br>Used validated measures, good sample size, multiple imputation for missing data	Yes
	**MMAT for quantitative RCT**<br>1. Is randomization appropriately performed?<br>Web-based randomization integrated into REDCap; stratified by site and cohort; allocation lists generated in advance by statistician	Yes
	2. Are the groups comparable at baseline?<br>Baseline characteristics (age, ethnicity, CORE-OM scores) were similar between groups (Table 1).	Yes
	3. Are there complete outcome data?<br>Follow-up rates dropped over time: 75% at 3 months, 64% at 7 months, 63% at 12 months. Missing data was handled via multiple imputation in sensitivity analysis.	Yes
	4. Are outcome assessors blinded to the intervention provided?<br>Data assessors and observational coders were masked to allocation. (Participants and interveners not possible to mask).	Yes
	5. Did the participants adhere to the assigned intervention?<br>Mean attendance was 6.4 out of 10 sessions. Adherence and attendance varied a lot.	CT

Study ID: Saïas 2013 *Reviewers initials: CFT/ KDB*

Reference:

Saïas, T., Greacen, T., Tubach, F., Romain Dugravier, Marcault, E., Tereno, S., Tremblay, R.E. and Guedeney, A. (2013). Supporting Families in Challenging contexts: the CAPEDP Project. Global Health Promotion, 20(2_suppl), pp.66–70. doi:https://doi.org/10.1177/1757975913483335.

Other publications from same study:

Guedeney, A., Dugravier, R., Guedeney, N., Tubach, F., Greacen, T., & Saias, T.. Prevention in Vulnerable Families: First Results of the French CAPDEP Study. Archives of Women's Mental Health, 2013a; 16(Suppl 1), S4–S5

Tereno S, Guedeney N, Dugravier R, Greacen T, Saïas T, Tubach F, Guédeney A. Implementation and assessment of an early home-based intervention on infant attachment organisation: the CAPEDP attachment study in France. Global health promotion. 2013 Jun;20(2_suppl):71-5.

Guedeney, A., Dugravier, R., Guedeney, N., Tubach, F., Greacen, T., & Saias, T. (2009). CAPEDP-Attachment: Parenting Skills and Attachment in Infants: Reducing Mental Health Risks and Promoting Resilience A French Project to Decrease Disorganized Attachment. Perspectives in Infant Mental Health, 17(1-2).

Tubach, F., Greacen, T., Saïas, T., Dugravier, R., Guedeney, N., Ravaud, P et al. (2012). A home-visiting Intervention Targeting Determinants of Infant Mental health: the Study Protocol for the CAPEDP Randomized Controlled Trial in France. BMC Public Health, 12(1). doi:https://doi.org/10.1186/1471-2458-12-648.

Guedeney A, Wendland J, Dugravier R, Saïas T, Tubach F, Welniarz B, et al. Impact of a randomized home-visiting trial on infant social withdrawal in the CAPEDP Prevention Study. Infant Mental Health Journal. 2013b; 34(6):594-601.

Guedeney A, Guedeney N, Tereno S, Dugravier R, Saias T, Tubach F, Wet al. Early prevention through a mental health programme for vulnerable families: preliminary findings and what we have learned in conducting the French CAPEDP study. InEarly parenting and prevention of disorder 2018 Apr 17 (pp. 73-89). Routledge.

Methods	**Study design**: Prospective randomised controlled trial (RCT) (abstract)<br>**Primary aim**: to assess the impact of a home-visiting program on child mental health (age 2), postnatal depression (3 months), and the quality of the home environment (12 months<br>**Intervention** (type + pregnancy/postnatal intervention): home-based, manualized but tailored intervention delivered by psychologists starting from the 3rd trimester of pregnancy (27 weeks) through to the child's second birthday<br>**Baseline measurements**: socio-demographics (age, education, income), pregnancy history, and EPDS scores<br>**Duration of study**: 2006 -2011.<br>**Ethics**: Approved by CPP Ile de France IV and CNIL.

Participants	**Setting**: 10 public hospitals, representing a broad cross-section of urban antenatal services in Northen part of city of Paris and suburbs. France.<br>**Inclusion criteria**: < 26 years old; (2) < 27 weeks pregnant; (3) Sufficient fluency in French; (4) At least one vulnerability factor: < 12 years education, low income (eligible for free healthcare), or planning to raise child alone<br>E**xclusion criteria**: planning to move away from the area; or already receiving sustained medical/social care for severe conditions (e.g., mental disorders like schizophrenia requiring long-term follow-up)<br>**Total number of participants**: **4**40 families randomized; intervention: 222; control: 218<br>**Mean age of participants (years) (SD) [min, max]:** 22.3 years<br>**Mean gestation age at time of intervention start:** Recruitment occurred before the 27th week of pregnancy (intervention began 7th month)<br>**Ethnicity**: 52.1% were first-generation immigrants<br>**Marital status:** 44.2% were living alone and single<br>**Socio-economic status**: high vulnerability; 46.8% eligible for government medical aid (welfare/low income); 34.9% considered themselves poor<br>**Education**: 83.9% had less than 12 years of schooling<br>**Pre-existing medical or mental health conditions:** 44.7% had prenatal depression scores (EPDS) > 11.9 at baseline<br>**Other lifestyle/health behaviors :** Not reported
Intervention	**Type and content of intervention** (Tidier if possible/reported): home visiting programme in addition to usual care.<br>*What*: intervention covering 39 topics (health, parenting skills, attachment security). It utilized video-feedback (filming daily interactions) to improve maternal sensitivity.<br>Included strict safety measures, home visitors had weekly supervision to detect danger. They were instructed to report to the main investigator immediately if they detected "suicidal thoughts" or psychopathology requiring specific support.<br>*Who provided*: qualified psychologists (specifically trained, non-nurses)<br>*Where*: participant's home<br>When and how much: 44 planned visits total. Weekly from 7th month of pregnancy; decreasing frequency until child is 24 months old<br>*Modifications*: tailored to family needs<br>*How well (if applicable)*: NA<br><br>**Type and content of control (if applicable, Tidier if possible/reported**): Usual care, as described above, involved free access to the PMI network and to community mental health services, free antenatal maternity screenings by local GPs, extensive social security allowances and facilitated access to housing.
Outcomes	**Outcome considered in the review and reported in the study:**<br>o No suicide attempts were observed in either the intervention or control group (Trial abstract, suicidality was not measured as a primary or secondary outcome.<br>o Intervention did not reduce maternal depression but was associated with increased use of well-baby clinics and mental health services in the intervention group.
Underpinning theory of the intervention	Grounded in a multidimensional theoretical framework that combined Bowlby's attachment theory, emphasizing the infant's need for a secure base, with Bronfenbrenner's ecological model, which views vulnerability as the accumulation of environmental risk factors. The intervention also drew heavily upon Bandura's self-efficacy theory to promote parental empowerment and Fraiberg's psychodynamic concepts, specifically "Ghosts in the Nursery," to help mothers understand how their past experiences might influence their current parenting behaviours. Additionally, the use of video feedback was theoretically supported by concepts of mentalization, aiming to improve the mother's capacity to reflect on her child's mental states.
Challenges & strategies, lessons learn	Challenges e.g. high attrition rates, with approx 36.8% of participants dropping out by three months postpartum and nearly 50% by 18 months, and diffficulties of establishing a statistical contrast due to France's generous "care as usual" system which offers free universal maternal support. To mitigate these issues, the team employed strategies such as flexible rescheduling, text message reminders, and maintaining telephone contact even when home visits were refused. While the decision to use trained psychologists rather than nurses allowed for a specialized focus on mental health determinants, these professionals

	sometimes lacked general perinatal health knowledge and reported feeling "unsettled" by the gap between the attachment-based theoretical goals and the families' urgent socioeconomic needs.<br>While video-feedback proved to be a powerful tool for enhancing maternal sensitivity, the intervention was most effective for specific subgroups, such as preventing social withdrawal in infants of non-depressed mothers, suggesting that future programs in similar welfare contexts may need to be more intensive or differently targeted to successfully assist the most clinically depressed and vulnerable women.
What is new? (conclusions)	Authors concluded that early prevention programs delivered by psychologists are feasible but must be carefully adapted to the local social welfare context, and that future interventions might need to be more intensive or different in nature to successfully impact the most high-risk, depressed mothers.
Additional references / Other comments	

**Quality Assessment – Saïas 2013**	*Reviewer initials: CFT/YDE*
Critical Appraisal	*The Mixed Methods Appraisal Tool (MMAT) 2018 will be used for quantitative, qualitative and mixed-methods studies.*<br><br>**Screening questions**<br>1. <u>Are there clear research questions?</u><br>The primary aims (child mental health, postnatal depression, home environment) are clearly defined in the protocol and main paper — Yes<br><br>2. <u>Do the collected data allow to address the research questions?</u> — Yes<br>Yes – the collected data is relevant to maternal mental health and infant attachment.<br><br>**MMAT for quantitative randomised studies**<br><br>1. Is randomization appropriately performed? — Yes<br>Computer-generated sequence, centralized by fax to ensure concealment.<br><br>2. Are the groups comparable at baseline? — CT<br>Main paper said no significant differences found in baseline characteristics or prenatal depression scores, but characteritcs not presented by study group<br><br>3. Are there complete outcome data? — No<br>High attrition. 36.8% dropout by 3 months postpartum; 50% missing data by 18 month<br><br>4. Are outcome assessors blinded to the intervention provided?<br>Evaluators were a separate team and blinded to assignment — Yes<br><br>5. Did the participants adhere to the assigned intervention?<br>The mean number of home visits completed was 24.5 (out of 44 planned) for those with available data at 18 months — CT

Study ID: Tachibana 2020	Reviewers initials: CFT/YDE

Reference:

Tachibana Y, Koizumi N, Mikami M, Shikada K, Yamashita S, Shimizu M, Machida K, Ito H. An integrated community mental healthcare program to reduce suicidal ideation and improve maternal mental health during the postnatal period: the findings from the Nagano trial. BMC psychiatry. 2020 Dec;20:1-5.

Other publications from same study:

Methods	**Study design**: non-randomized controlled trial.<br>**Primary aim**: to reduce postnatal suicide ideation and improve maternal mental health.<br>**Intervention** (type + pregnancy/postnatal intervention): population based maternal suicide prevention program (postnatal intervention): mental health screening to detect women with suicidal ideation or who were at risk of psychosocial problems; and clear referral and management pathway for managing those women.<br>**Baseline measurements**: mother's age, father's age, siblings (number, sex, age), method of birth, birth weight. No baseline EPDS/mental health scores recorded.<br>**Duration of study**: 2015-2016
Participants	**Setting**: rural areas, urban areas, and industrial areas of Nagano city, Japan<br>**Inclusion criteria**: sole inclusion criterion was submission of a birth notification to the Nagano city government – those identified at risk were followed up by public health nurses. No exclusion criteria.<br>**Total number of participants**: 464 women: 230 allocated to the control group (submitted their birth notification forms to the Nagano city's local government from Nov 2015 to Mar 2016) and 234 allocated to the intervention group (submitted their forms between Apr 2016 and Jul 2016)<br>**Mean age of participants (years) (SD) [min, max]:** 31.98 (4.85) [17, 49] in intervention group; 31.82 (5.18) [17, 43] in control group.<br>**Mean gestation age at time of intervention start:** N/A.<br>**OR months after birth at time of intervention start:** Follow up after birth notification within 4 months.<br>**Ethnicity**: Not reported (assumed Japanese)<br>**Marital status**: Not reported<br>**Socio-economic status**: Not reported<br>**Education**: Not reported<br>**Pre-existing medical or mental health conditions:** Not reported<br>**Other lifestyle/health behaviors (i.e.** substance misuse, physical/sexual abuse; suicide attempts in lifetime): Not reported
Intervention	**Type and content of intervention** (Tidier if possible/reported): usual care plus maternal suicide prevention program<br>*What*: continuum and multidisciplinary maternal and child health service: 1) the multidisciplinary maternal and child health service team that shared the referral and management protocol processes 2) the multidisciplinary maternal and child health team in case the public health nurses or the other professionals related to mother and child noticed psychosocial risks with the family, 3) case formulation and management for women with suicidal ideations (including psychoeducation)<br>*Who provided*: A multidisciplinary clinical network based on NICE guidelines. The clinical network involved mother and child health service hospitals and institutes in Nagano city, and consisted of multidisciplinary perinatal service professionals including gynaecologists, midwives, nurses, public health nurses (who did the home visits), paediatricians, social workers, and psychiatrists under the support of the Nagano city public health centre and the Nagano City Medical Association.<br>*How*: All the postnatal women received a mental health screening with the EPDS by the public health nurses who did home visit in the neonatal periods. The public health nurses administered the self-harm question of the EPDS, which is a question related to suicide ideation, also noting any past psychiatric history of schizophrenia, major depression, or bipolar disorder (note the self-harm question of the EPDS has been used for assessing suicidal ideation in the perinatal period and is considered valid). They also assessed the mothers' mental health using the total score of the EPDS.<br>In instances where the public health nurse identified a mother as exhibiting suicide ideation, the TALK steps (i.e., Tell, Ask, Listen, Keep safe) were performed for their psychological crisis interventions for that visit. The public health nurses assessed psychosocial risk comprehensively including suicidal risk and offered psychoeducation for women and their family, if possible, based on the results of the assessments (i.e. risk of narrow-mindedness when they have suicidal ideation and their tendency to be isolated with sense of hopelessness; necessity of psychological or psychiatric care to relieve their suicidal ideations; medications can also be effective; discussed family support conditions and collaborated with family members, if possible; urged mothers to promise not to harm themselves or their children and agreed upon a"SOS" signal that

	could be directed toward specialists such as psychiatrists or public health nurses in case of a mental health crisis. In cases that the women's families were concerned with or exhausted with their cares, the public health nurses also heard attentively and empathically, they respect their families' opinions and think together how they should manage the problem.<br><br>Part-time psychiatrists of the public health centres provided expert advice on the risks and benefits of psychotropic medication use during pregnancy and breastfeeding for the mothers with whom they consulted. Related mother and child health service professionals in Nagano city followed the referral and management protocols for services across all levels of the existing stepped-care frameworks for maternal mental health problems, problems related to childcare or the family environment, and child protection.<br><br>Follow-up sheets were also developed to target case formulation and support women at risk of psychosocial problems. The follow-up sheet empowered public health nurses, who are non-psychiatric professional to consistent assess mental health problems and follow-up with mothers at risk. Case formulation is an important component of intervention for suicide prevention. The contents of the follow-up sheets enabled case formulation and helped the nurses better understand the biopsychosocial aspects, of both the mother and child, systematically and informed them about care plan development.<br><br>*Where*: Home visits (nurses) /public health centers (psychiatrist) /unclear place of multidisciplinary meetings.<br>When and how much: Mothers' mental health assessed at the 3- to 4-month infant medical examination (T1) and the 7- to 8-month infant medical examination (T2) using the total score of the EPDS.<br>*Tailoring*: the intervention was designed based on previous intervention program (Suzaka trial), preliminary surveys, clinical guidelines for perinatal mental health, and discussions among the researchers and with healthcare professionals in study regions.<br>*Modifications*: Not reported<br>*How well (if applicable)*: authors report they closely monitored intervention intensity, described the narrative intervention process in detail, and investigated the levels of exposure to interventions in the post-intervention survey (but not specifically reported in this paper).<br><br>**Type and content of control (if applicable, Tidier if possible/reported)**: usual care alone.<br>*What*: home visits without mental health screening<br>*Who provided*: public health nurses<br>*How*: not reported<br>*Where*: public health centres<br>When and how much: Mothers' mental health assessed at the 3- to 4-month infant medical examination (T1) and the 7- to 8-month infant medical examination (T2) using the total score of the EPDS.<br>*Tailoring*: not reported<br>*Modifications*: not reported<br>*How well (if applicable)*: not reported
Outcomes	**Outcome considered in the review and reported in the study:**<br>Suicidal ideation<br>Suicidal Ideation was defined specifically as a score >0 on Item 10 of the EPDS (Self-harm question)<br>Suicidal ideation was significantly lower in the intervention group compared with the control group at T1 (p = 0.014); however, this significant between-group difference did not continue to T2 (p = 0.111).<br>The results of the main and sensitivity analysis for the self-harm question of the EPDS and the total score of the EPDS are considered to be consistent<br>Women's general mental health<br>Intervention effects on maternal mental health were measured using the total score of the EPDS, which was significantly improved in the intervention group compared with the control group at T1. Here, the significant difference continued to T2 (p = 0.049).
Underpinning theory of the intervention	Authors think the effectiveness mechanism of the present intervention program partly derived from the same mechanism of our previous intervention program (the Suzaka trial, see below). The previous intervention program used the EPDS interviews conducted by the public health nurses, which played the following roles: 1) opening up the conversation about psychosocial issues; 2)raising awareness and educating pregnant women about the various psychiatric and psychosocial conditions; and 3) developing good relationships with the women by inquiring about their psychosocial problems. These allow the establishment of trust, open and frank information receiving, in turn, to a better understanding of the needs of the expectant women, which led to early intervention if they needed, and their better access to the maternal and child health services.

	In addition, the intervention program had a characteristic approach that involved using case management intervention for women with suicidal ideations. Case management is a comprehensive psychosocial care approach performed according to the patient's individuality and has been shown to be effective in particularly complex cases. Continuum case managements based on psychiatric and psychosocial assessments has been shown to prevent recidivation of suicide attempts and self-harm. The follow-up system of this study's intervention matched Action-J's case management components: 1) crisis intervention performed by public health nurses when deemed necessary, 2) the comprehensive assessment of psychosocial risk by public health nurses using the EPDS and "follow-up sheet", and if the nurses noted possible suicidal ideation with the women, they confirmed its existence; 3) psychoeducation performed by public health nurses to manage suicidal ideation and give advice on seeking help and using help resources for women with suicidal ideation; and 4) meetings by a multidisciplinary professional team to discuss how to support these women, with the team including public health nurses and mental health professionals (i.e. psychiatrists and psychologists) performing follow-up. Since Action-J's case management system has evidence supporting the prevention of repeat suicide attempts and self-harm, authors believe that the significant reduction in suicide ideation according to the EPDS in the intervention group compared to the control group may be due to the application of the case management system in this program.<br><br>Also, the main providers were public health nurses who also cared for the mothers and their children. The results indicate the effectiveness of public health nursing supports for reducing maternal suicidal ideation and improving maternal mental health. Public health nurses and other related professionals can collaborate and administer material and child healthcare through a multidisciplinary clinical network. The intervention also provides public health nurses with a routine mental health screening system, and a systematic follow-up sheet for mental healthcare. Thus, their role is pivotal for ensuring adequate and community-based maternal mental healthcare. If nurses perform integrated mental healthcare, like routine mental health screening, collaborate with other professionals, and follow-up with mothers and children using systematic assessments and care plans, their maternal mental health interventions will likely be very effective.
Challenges & strategies, lessons learn	Very little reported on experiences challenges or lessons learn other than strengths and limitations.<br>The intervention does not require extra human resources or facilities and can be performed by adding maternal mental healthcare and suicidal ideation management to usual care practices for mothers and their children; clear referral and management pathways; use of clinical network that comprises a specialist multidisciplinary local perinatal service, which provides direct services, consultation, and advice within the context of maternity services, other mental health services, and community series.<br>But suicidal ideation in the postnatal period was assessed using EPDS and clinical interviews by public health nurses (no phyquiatrics); validity of EPDS self-harm question; effectiveness in decreasing suicidal ideation in the population sample, but not possible to determine whether the program decrease the number of postnatal suicides; no baseline assessments of EPDS scores before the intervention; authors cannot suggest the degree to which the seasonality of the participants' deliveries affected the results.<br>The distribution of the answers to the self-harm question of the EPDS, for the intervention and the control groups, indicate this intervention approach may be effective for mothers with low to moderate suicidal ideation. The frequency of "hardly ever" and "sometimes" as response choices to the self-harm question of the EPDS were low in the intervention group, compared with the control group. However, the frequency of the answer "yes, quite often" was the same between the two groups. Since the frequency of response "yes, quite often" in the self-harm question of the EPDS is very rare, this study lacks evidence to infer the effects of the program on those who have severe suicidal ideation.
What is new? (conclusions)	The results indicate that this program may reduce maternal suicidal ideation at 3–4 months postnatally and improve women's mental health during the postnatal periods of 3–4 to 7–8 months. A postnatal mental health package that includes suicide ideation assessments and a case management approach, like this program, should be included as an important component of general postnatal care.<br>Further studies, including RCTs using the present intervention program,are needed.
Additional references / Other comments	Tachibana Y, Koizumi N, Akanuma C, Tarui H, Ishii E, Hoshina T, Suzuki A, Asano A, Sekino S, Ito H. Integrated mental health care in a multidisciplinary maternal and child health service in the community: the findings from the Suzaka trial. BMC pregnancy and childbirth. 2019 Dec;19:1-1.<br><br>Since no women who were followed up by the public health nurses due to expressing suicidal ideation within the overlap period of the control and the intervention group (i.e. April 2016), the suicide prevention intervention of the present program was not performed for the control group participants in that period.

**Quality Assessment – Tachibana 2020**	*Reviewer initials: CTF/YDE*
Critical Appraisal	*The Mixed Methods Appraisal Tool (MMAT) 2018 will be used for quantitative, qualitative and mixed-methods studies.*<br><br>**Screening questions**<br>1. <u>Are there clear research questions?</u> — Yes<br>Ro investigate if this programme could reduce suicidal ideation and improve maternal mental health.<br><br>2. <u>Do the collected data allow to address the research questions?</u> — Yes<br>Yes data collected on baseline characteristics and limits to use of EPDS (self-harm question)<br><br>**MMAT for quantitative non-randomised studies**<br>1. <u>Are the participants representative of the target population?</u> — Yes<br>Yes, population-based intervention, the self-harm question of the EPDS could identify targeted population, which were mothers at increased risk of suicide.<br><br>2. <u>Are measurements appropriate regarding both the outcome and intervention (or exposure)?</u> — Yes<br>Yes, but limited evidence on use of EPDS (self-harm question) to assess suicidal ideation. Unclear how the different measures of the intervention/exposure affect the outcomes<br><br>3. <u>Are there complete outcome data?</u> — Yes<br>Yes, missing data less than 10% in both groups<br><br>4. <u>Are the confounders accounted for in the design and analysis?</u> — CT<br>The study reports that "seasonal confounding factors" may have affected results because the control and intervention groups gave birth in different seasons. Additionally, the lack of baseline EPDS scores and missing socioeconomic data make it impossible to rule out confounding.<br><br>5. <u>During the study period, is the intervention administered (or exposure occurred) as intended?</u> — CT<br>Authors report closely monitoring of intervention intensity, described the narrative intervention process in detail, and investigated the levels of exposure to interventions in the post-intervention survey (but not specifically reported in this paper).

**Study ID: Wilkinson 2017**	*Reviewers initials: CFT/YDE*
**Reference:**<br>Wilkinson A, Anderson S, and Wheeler SB. Screening for and treating postpartum depression and psychosis: a cost-effectiveness analysis. Matern Child Health, 2017: 903–914	
**Other publications from same study:**	
Methods	**Study design**: Cost-effectiveness modelling study (decision tree)<br>**Primary aim**: to model the cost-effectiveness of screening and treating postpartum depression (PPD) and postpartum psychosis (PP) compared to no screening<br>**Intervention** (type + pregnancy/postnatal intervention): Routine screening for postpartum depression and postpartum psychosis<br>**Baseline measurements**: As this was a modelling study using a hypothetical cohort, there were no baseline measurements from participants however the model incorporated input parameters from previously published literature from between 1995 and 2015. This included prevalence of postpartum depression and psychosis, detection and treatment rates under usual care and utility values for quality-of-life adjustments.<br>**Duration of study**: The model was populated using data obtained from a review of literature published between 1995 and 2015; rhe model itself simulated a 2-year time postpartum period<br>**Ethics**: Hypothesised cohort, exempt from Institutional Review Board.

Participants	**Setting**: This model was developed using a US Medicaid Healthcare context, in North Carolina, USA<br>**Inclusion criteria**: This study consisted of a hypothetical cohort of 1000 Medicaid-insured women of reproductive age (18–49 years) who had experienced one birth in the past year.<br>**Total number of participants**: 1000<br>**Mean age of participants (years) (SD):** Range from 18-49.<br>**Mean gestation age at time of intervention start:** Not reported.<br>**OR months after birth at time of intervention start:** n/a<br>**Ethnicity**: Not collected.<br>**Marital status**: n/a<br>**Socio-economic status**: Hypothetical cohort represented low-income women as they were all assumed to be covered under Medicaid, a federal programme providing healthcare for individuals with limited income.<br>**Education**: n/a<br>**Pre-existing medical or mental health conditions:** n/a<br>**Other lifestyle/health behaviors**/risk factors? n/a
Intervention	**Type and content of intervention** (think Tidier if possible/reported?)<br>*What*: Modelled strategy for routine screening and treatment of postpartum depression and postpartum psychosis during outpatient visits. Screening was conducted using the Edinburgh Postnatal Depression Scale (EPDS).<br>*Who provided*: This model assumed that women would be screened for postpartum depression and psychosis by clinical physicians who had access to psychiatrist mentorship.<br>*How*: Women who screened positive were offered evidence-based treatments including pharmacological and psychotherapeutic interventions. Treatment options included fluoxetine, interpersonal therapy (IPT) or a combination of both.<br>*Where*: The intervention was modelled as taking place in outpatient healthcare settings under Medicaid coverage which reflects typical service delivery for low income women in the United States.<br>When and how much: 2 years<br>*Tailoring*: fluoxetine chosen based on cost and safety for breastfeeding<br>*Modifications*: n/a<br>*How well (if applicable)*: n/a<br><br>**Type and content of control (if applicable):**<br>*What*: Usual care, assuming low detection and treatment rates without screening. Nothing else reported
Outcomes	**Outcome considered in the review and reported in the study:**<br>**Suicide:**<br>Usual Care: themodel predicted 0.58 suicides.; Intervention: themodel predicted 0.34 suicides.<br>Difference: theintervention resulted in a reduction of 0.24 suicides per 1,000 women compared to usual care --- This is a decision-tree mathematical model thus, these figures represent the projected absolute reduction based on probabilities derived from existing literature, where the model assumes that screening leads to higher treatment rates, and treatment reduces the risk of suicide.<br>* Suicide /ideation: while the model included suicidal ideation (assumed in 10% of cases) as a factor that lowers quality of life (utility scores), the specific outcome reported in the final results table 4 was the number of completed suicides prevented.<br><br>**Other**: Compared to usual care, implementation of screening and treatment resulted in 29 more (hypothetical) women achieving remission from postpartum depression or postpartum psychosis over the 2-year postpartum period. Quality-adjusted life-years (QALYs) were also increased by an additional 21.43 compared to 'usual care'.<br>Costs increased by almost $300k with intervention implementation.
Underpinning theory of the intervention	While the study does not present a theory of change, the assumed rationale is based on using early detection to increase earlier intervention and thus improve clinical outcomes, save costs and address under-detection of perinatal mental illness within low-income populations.
Challenges & strategies, lessons learn	Due to data limitations, the study relied on some international sources for treatment effectiveness which affects generalisability to the U.S Medicaid population. In addition, this is a modelling study that presumes data rather than actual measurements which limits the certainty of the results. The study also does not account for variations in risk factors within a cohort such as comorbidities, social challenges and past mental health. The hypothetical cohort use makes it less reliable.

What is new? (conclusions)	This is one of the first studies to model the cost-effectiveness of screening and treating both postpartum depression and postpartum psychosis in a Medicaid-insured population. It provides new evidence quicker and the use of theoretical cohorts may be of use for future research.<br>The study concludes that implementing routine screening and treatment for postpartum depression and psychosis is likely to be cost-effective within a Medicaid healthcare setting. This is a start for future research material.
Additional references / Other comments	

**Quality Assessment – Wilkinson 2017**	*Reviewer initials: CFT/YDE*
Critical Appraisal	*The Mixed Methods Appraisal Tool (MMAT) 2018 will be used for quantitative, qualitative and mixed-methods studies.*<br><br>**Screening questions**<br>1. <u>Are there clear research questions?</u><br><br>Yes - evaluating the cost-effectiveness of screening on postpartum depression and psychosis detection and treatment compared to usual care<br><br>2. <u>Do the collected data allow to address the research questions?</u><br><br>Original data was not collected, and outcomes were based on modelled estimates from previously published literature.<br><br>*MMAT is not designed for economic modelling studies/hypothetical cohorts. MMAT tool cannot be used to assess hypothetical cohorts in the same way as empirical research.*

Records of ongoing studies (5)

1) NCT05852314: A Problem-Solving Intervention for Women With Suicidal Ideation During Postnatal Period in Pakistan

2) Sattar R, Husain N, Kiran T, Eylem O, Chaudhry N. Culturally Adapted Problem-Solving Intervention for Women Experiencing Suicidal Ideation During Postnatal Period. BJPsych Open. 2024 Jun;10(S1):S24

3) NCT06208293: Opportunities for Suicide Prevention Integration Into Task-shifted Mental Health Interventions in Low-resourced

4) NCT06849869: Collaborative Care Model for Perinatal Depression Support Services -- Population-Level Equity-Centered Systems Change (COMPASS-PLUS): a Hybrid Type 2 Cluster Randomized Trial

5) NCT07011940: Walk With Me (WWM) for Perinatal Grief.

Records of studies awaiting classification (9)

1) NCT06666400 Living Healthy for Moms RCT (LHMoms)

2) NCT07104123 Substance Use in Pregnant People - Optimizing Retention in Treatment by Maximizing Opportunities for Management

3) NCT06770244: Positive Psychology-Based Intervention Program for Postpartum Depression Within Primary Healthcare System: Protocol for a Randomized Controlled Trial.
Fan Z, Weng M, Su J, et al. Positive psychology-- based intervention programme for postpartum depression among pregnant women within the primary healthcare system: protocol for a randomised controlled trial. BMJ Open 2025;15:e101115. doi:10.1136/ bmjopen-2025-101115

4) NCT06968390: Perinatal Accelerated iTBS Neuromodulation Therapy

5) NCT07027761 Maternal Mental Health Intervention (MaMHI) Study: A Combined Non-inferiority and Superiority Randomized Controlled Trial Comparing the Clinical and Cost-effectiveness of an Online Self-help Intervention and a Brief Face-to-face Intervention Versus Usual.

6) NCT06544941 Improving Maternal Mental Health in Military-Affiliated Pregnant Women: Effectiveness of a Smart Bassinet.
Okun ML, Payne JL, Osborne LM, Feliciano L, Lac A. Effects of Using a Smart Bassinet on the Mental Health of Military-Affiliated Pregnant Women: Protocol for a Randomized Controlled Sleep Health and Mood in Newly Expectant Military Mothers (SHINE) Trial. JMIR Research Protocols. 2025 Apr 10;14(1):e66439

7) NCT07025902 Holistic Support Pathways for Maternal Psychological Wellbeing: A Comprehensive Approach From Preconception to Postpartum (SOS-Mamma).

8) NCT06442774 Digital Tool to Improve Maternal Mental Health: Enhancing Well-being, Early Detection, Diagnosis Support, and Monitoring of Mental Health Problems During the Perinatal Period

9) NCT07168642 A Type I Hybrid Effectiveness-Implementation Trial of Mothering From the Inside Out (MIO)
